# Integrative bioinformatics analysis of potential convergent evolution in tardigrade stress-response proteins

**DOI:** 10.1007/s44154-026-00286-5

**Published:** 2026-03-23

**Authors:** Sawar Khan, Ayesha Nisar, Abdul Qadeer, Sijie Liu, Sardar Azhar Mehmood, Lingqiang Zhang, Zanxian Xia

**Affiliations:** 1 Department of Cell Biology, School of Life Sciences, Central South University, Changsha, 410013 China; 2 Institute of Molecular Biology and Biotechnology, The University of Lahore, Lahore, 54000 Pakistan; 3 Key Laboratory of Genetic Evolution & Animal Models, Kunming Institute of Zoology, Chinese Academy of Sciences, Kunming, Yunnan 650201 China; 4 Department of Zoology, Hazara University, Mansehra, 21300 Pakistan; 5 State Key Laboratory of Medical Proteomics, Beijing Proteome Research Center, National Center for Protein Sciences (Beijing), Beijing Institute of Lifeomics, Beijing, 102206 China

**Keywords:** Tardigrades, Intrinsically disordered proteins, SAHS, CAHS, MAHS, Dsup

## Abstract

Tardigrades, known for their extraordinary resilience to extreme environmental conditions, employ specialized stress-response proteins to maintain cellular integrity under stress. These proteins, including SAHS, CAHS, MAHS, and Dsup, play critical roles in protecting cells from dehydration, radiation, and other environmental stressors. Previous work has characterized many of these proteins in *Ramazzottius varieornatus*, but the taxonomic breadth, copy-number variation, and structural diversity of these families across other tardigrade lineages remain poorly understood. The recent expansion of genomic resources for additional species motivated a comprehensive multi-species analysis to map ortholog diversity and structural features across tardigrade taxa. We investigated the genetic, structural, and evolutionary features of these stress-response proteins using an integrative bioinformatics approach, analyzing gene structure, orthologous clustering, molecular phylogeny, conserved motifs, and 3D protein structures across four tardigrade species—*R. varieornatus, Hypsibius exemplaris, Paramacrobiotus metropolitanus,* and *H. henanensis*. Our results reveal significant variation in predicted gene copy numbers and sequence conservation that reflect species-specific adaptations to environmental stress. Orthologous clustering showed shared evolutionary patterns across species, and conserved motifs were identified within the SAHS, CAHS, and MAHS families. Notably, we identified a new Dsup protein (H.Henanensis.Chr5.66), a potential ortholog in *H. henanensis*, thereby expanding the known diversity of Dsup proteins. The Dsup protein family exhibited notable sequence diversity across species, yet structural analyses revealed conserved α-helix regions and hydrophobicity patterns, suggesting a flexible conformation that aids DNA protection during extreme stress. Phylogenetic analyses revealed patterns consistent with parallel clustering patterns among stress-response proteins. This study advances our understanding of the molecular adaptations that underpin tardigrades' resilience and offers insights into potential applications for enhancing stress tolerance in other organisms.

**Supplementary Information:**

The online version contains supplementary material available at 10.1007/s44154-026-00286-5.

## Introduction

Tardigrades, microscopic ecdysozoan invertebrates, represent a remarkable group of extremophiles capable of surviving in some of the harshest environments on Earth (Jönsson 2019; Li et al. 2024; Møbjerg et al. 2011). With over 1,500 described species, tardigrades exhibit a cosmopolitan distribution, inhabiting diverse ecosystems, including freshwater, marine, and terrestrial biomes across the globe (Fleming et al. 2024). Their resilience is unparalleled, with different species exhibiting varying forms of extremotolerance, enabling them to withstand desiccation, extreme temperatures, high pressure, ionizing radiation, and even the vacuum of space (Arakawa 2022; Guidetti et al. 2011). This extraordinary survival ability is largely attributed to specialized molecular and cellular adaptations, which allow tardigrades to endure and recover from extreme stress conditions (Giovannini et al. 2022; Hashimoto et al. 2016).

At the core of tardigrades' ability to tolerate extreme stress are unique stress-response proteins, which play a crucial role in cellular protection. Among these, Secretory Abundant Heat-Soluble (SAHS), Cytoplasmic Abundant Heat-Soluble (CAHS), Mitochondrial Abundant Heat-Soluble (MAHS), and Damage Suppressor (Dsup) proteins have been identified as key players in resisting dehydration, radiation, and other environmental stressors (Boothby et al. 2017; Hashimoto et al. 2016; Hesgrove and Boothby 2020; Tanaka et al. 2015; Yamaguchi et al. 2012). The discovery of these proteins, particularly in *Ramazzottius varieornatus*, revealed the presence of a distinct group of intrinsically disordered stress-response proteins, which contribute to stress resilience through various protective mechanisms (Arakawa 2022; Fukuda and Inoue 2018; Fukuda et al. 2017; Yamaguchi et al. 2012). In recent years, tardigrade intrinsically disordered proteins (TDPs) have garnered significant research interest in genetics, molecular biology, cellular biology, and biophysics, with studies focusing on their structural and functional properties (Boothby et al. 2017; Chavez et al. 2019; Hashimoto et al. 2016; Malki et al. 2022; Tanaka et al. 2022).

Beyond fundamental biology, these proteins hold considerable biotechnological potential due to their exceptional protective properties. Research suggests that TDPs could be harnessed for applications in medicine, biotechnology, bioremediation, and space exploration, with potential uses in creating stress-resistant organisms, radiation and chemotherapy protection, stabilizing biomolecules for pharmaceuticals, and enhancing vaccine storage and transportation (Afshinnekoo et al. 2020; Furukawa et al. 2020; Hashimoto and Kunieda 2017; Jönsson 2019; Malki et al. 2022; Packebush et al. 2023; Tanaka et al. 2022; Tikhonova et al. 2022; M. Zarubin et al. 2024a, b; Zarubin et al. 2023).

A key concept in evolutionary biology is convergent evolution, where similar traits arise independently in unrelated lineages due to shared selective pressures. In the case of tardigrade stress-response proteins, SAHS, CAHS, MAHS, and Dsup appear to be uniquely evolved within tardigrades, yet they exhibit functional convergence with other protective proteins found in extremophilic organisms (Arakawa 2022; Boothby et al. 2017; Chavez et al. 2019; Yoshida et al. 2022). These proteins share common molecular strategies that safeguard against dehydration, radiation, oxidative damage, and other environmental challenges, offering insight into the broader principles of stress resilience.

Despite increasing interest in these stress-response proteins, much of our understanding is limited to a few species, primarily *Hypsibius exemplaris* and *R. varieornatus* (Arakawa 2022; Fukuda and Inoue 2018; Fukuda et al. 2017; Yamaguchi et al. 2012). While some tardigrades possess multiple copies of these protein-coding genes, the full extent of their diversity remains unclear, particularly for Dsup and MAHS families, which contain only a few known members (Chavez et al. 2019; Hashimoto et al. 2016; Zarubin et al. 2023). With the recent expansion of tardigrade genomic resources, including our own assembly **(**Li et al. 2024**)**, additional gene-family members are likely to be discovered, yielding a more comprehensive picture of their diversity and providing a foundation for future functional studies into mechanisms such as nuclear localization, DNA-binding specificity, and protein–protein interactions in stress responses. In this context, this study was designed to capitalize on the availability of high-quality, well‐annotated genomic data from *R. varieornatus, H. exemplaris, Paramacrobiotus metropolitanus*, and *H. henanensis*. By concentrating on these species—including underexplored taxa such as *H. henanensis* and *P. metropolitanus*—we aim to perform an in‐depth, integrative analysis of stress‐response protein evolution. This targeted approach facilitates a detailed investigation of gene structure, motif conservation, and structural properties of these proteins.

To this end, we performed comparative genome-wide and proteome-wide analyses of the four tardigrade species to investigate the genetic and structural characteristics of the SAHS, CAHS, MAHS, and Dsup protein families. Our approach included gene structure analysis, orthologous clustering, phylogenetic relationships, motif identification, and 3D structural modeling, aiming to uncover both conserved and divergent features that contribute to tardigrades’ extreme stress resilience. Notably, we identified a novel Dsup protein (H.Henanensis.Chr5.66) in *H. henanensis*, expanding the known diversity of this protein family and offering new insights into their evolutionary conservation. By elucidating the evolutionary and structural dynamics of tardigrade stress-response proteins, our findings contribute to a deeper understanding of molecular adaptation in extremophiles. Furthermore, these insights open new avenues for biotechnological applications, reinforcing the potential of TDPs in stress-protective innovations across various fields.

## Results

### Gene structure analysis of stress-response proteins in tardigrades

**Fig. 1:**
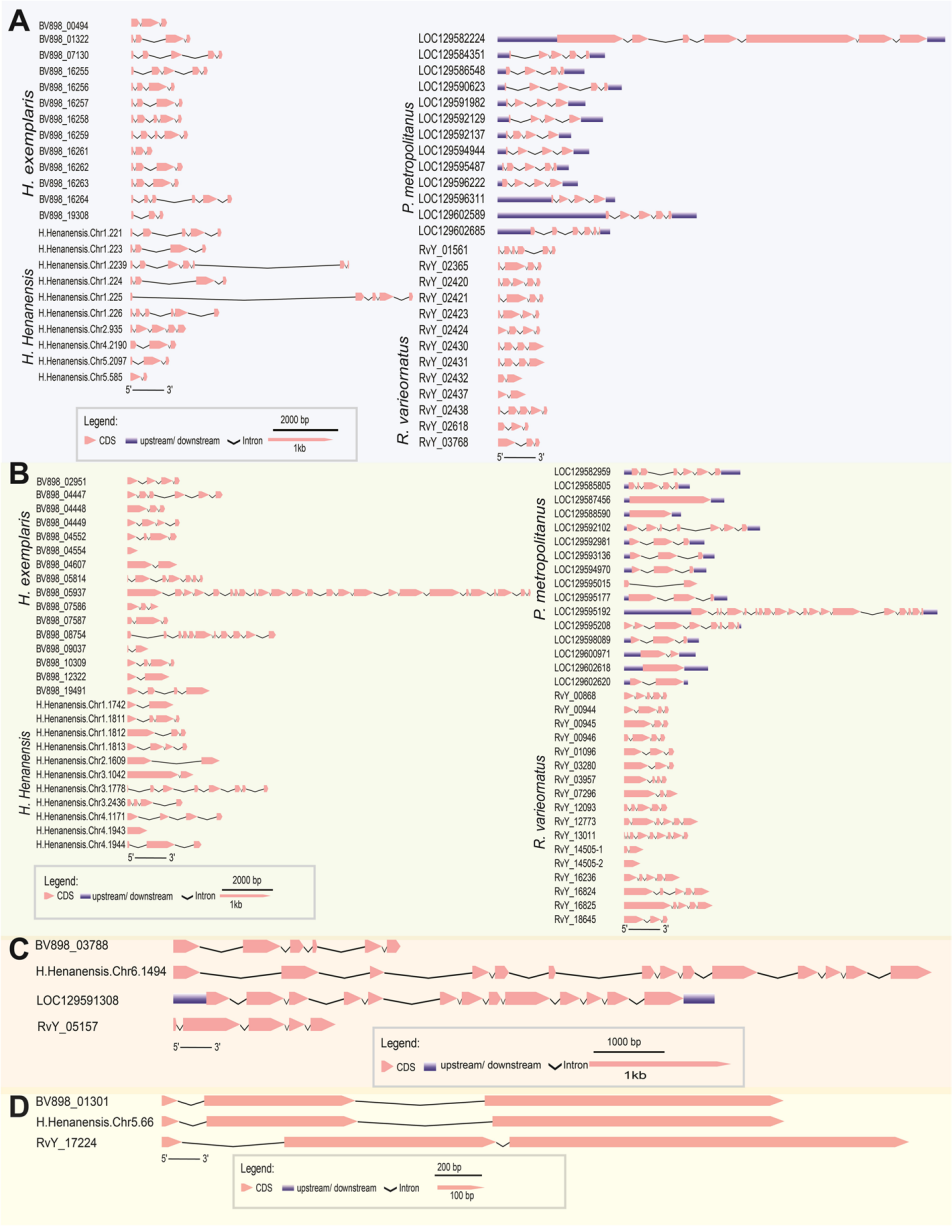
Gene structure analysis of stress-response proteins in tardigrades. **A** Gene structure of SAHS. **B** Gene structure of CAHS. **C** Gene structure of MAHS. **D** Gene structure of Dsup. Locus tags correspond to: RvY, *R. varieornatus*; BV898, *H. exemplaris*, LOC, *P. metropolitanus*; and H.Henanensis.Chr, *H. henanensis*

The gene structures of four major stress-response protein families—SAHS, CAHS, MAHS, and Dsup—were thoroughly analyzed across multiple tardigrade species, including *R. varieornatus*, *H. exemplaris*, *P. metropolitanus*, and *H. henanensis *(Fig. 1*)*. For the SAHS family, exon sizes spanned 1.40 kb (0.10–1.50 kb), while intron lengths spanned 6.40 kb (0.10–6.50 kb) (Fig. 1A). These gene structures varied somewhat among species, highlighting the adaptive diversification within the family. The CAHS family showed exon lengths spanning 1.50 kb (0.10–1.60 kb) and introns spanning 2.10 kb (0.10–2.20 kb) (Fig. 1B). MAHS genes exhibited relatively shorter exons spanning 0.30 kb (0.10–0.40 kb) and introns spanning 1.10 kb (0.10–1.20 kb) (Fig. 1C). Lastly, Dsup loci displayed exon lengths spanning 0.78 kb (0.10–0.88 kb) with introns spanning 0.40 kb (0.10–0.50 kb) (Fig. 1D).

Further analysis revealed species-specific variations in the number of genes encoding these stress-response proteins. For the SAHS family, *R.* varieornatus, *H. exemplaris* and *P. metropolitanus* each possessed 13 copies of SAHS-like sequence. In contrast, *H. henanensis* had a slightly lower number, with 10 copies. A similar trend was observed in the CAHS family, where *H. exemplaris* and *P. metropolitanus* contained 16 genes, and *R. varieornatus* had the highest count at 17 genes. *H. henanensis*, however, contained 11 genes, indicating a slight divergence in gene retention within this family. The MAHS family demonstrated a more conserved distribution, with each species possessing a single copy of the gene. This consistency suggests a potential functional constraint that limits the expansion of MAHS genes across tardigrades. In contrast, the distribution of Dsup-like sequences showed notable differences among species. While Dsup-like sequences were identified in *R. varieornatus, H. exemplaris*, and *H. henanensis*, no homologous sequences were detected in *P. metropolitanus*. The absence of a Dsup-like sequence in *P. metropolitanus* may indicate species-specific differences in DNA damage protection mechanisms, potentially reflecting alternative molecular strategies for stress tolerance in this species. These findings provide valuable insights into the evolutionary diversification of stress-response proteins in tardigrades. The observed variations in gene copy number and sequence conservation suggest distinct evolutionary pressures shaping the genomic architecture of these proteins, likely in response to species-specific ecological and environmental challenges.

### Orthologous cluster analysis of SAHS and CAHS

**Fig. 2:**
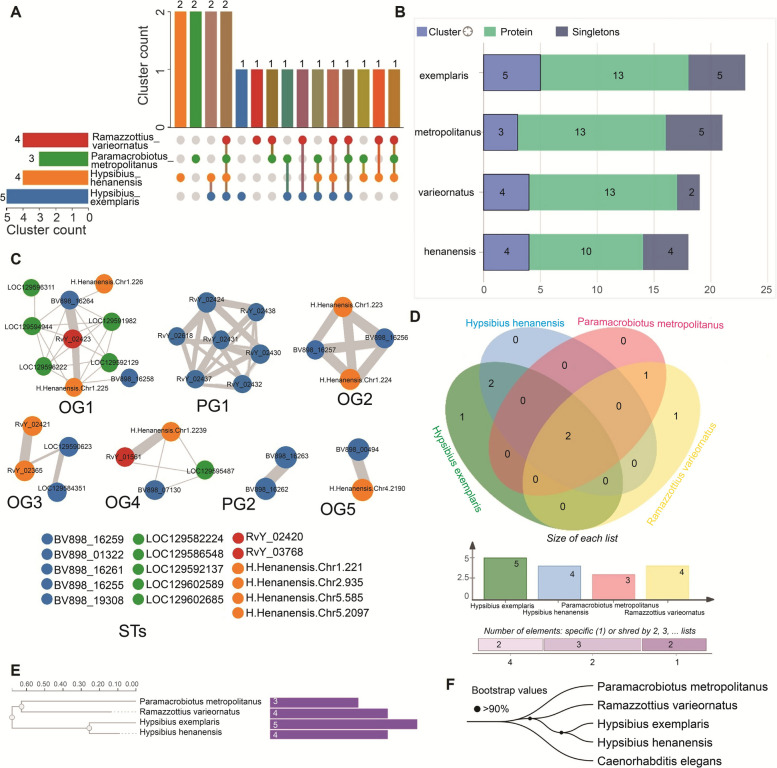
Analysis of orthologous clusters for SAHS proteins across the tardigrade species. **A** The UpSet plot illustrates the unique and shared orthologous clusters for SAHS proteins across species. The horizontal bar chart on the left depicts the count of orthologous clusters for each species, while the vertical bar chart on the right shows the number of clusters shared between species. The intersecting lines represent the overlapping sets. **B** Distribution of clusters, proteins, and singletons for SAHS proteins across the species. **C** Conserved ortholog groups (OGs), paralog groups (PGs), and singleton members (STs) of SAHS proteins in tardigrades. Edges widths represent numbers of interactions between the genes. **D** Venn diagram of cluster sharing for SAHS proteins across the species. **E** Gene tree of phylogenetic relationships based on the identification of highly conserved single-copy sequences of SAHS proteins across the tardigrade species. **F)** Concatenated species tree inferred from conserved proteins across the species. Locus tags correspond to: RvY, *R. varieornatus*; BV898, *H. exemplaris*, LOC, *P. metropolitanus*; and H.Henanensis.Chr, *H. henanensis*

For SAHS, the 49 proteins were distributed into a total of 7 clusters and 16 singletons (Fig. 2A-B). The identified clusters were further differentiated into 5 conserved orthologous groups (OGs) and 2 paralogous groups (PGs) of SAHS (Fig. 2C). One of the OGs was a single-copy cluster. The singletons accounted for 32.65% of the total SAHS proteins across species. *H. exemplaris* and *P. metropolitanus* contained 5 singletons each, whereas *H. henanensis* and *R. varieornatus* had 4 and 2 singletons, respectively. This high percentage of singletons suggests that these proteins might undergo lineage-specific adaptations in response to unique environmental pressures. While a high percentage of singletons could suggest lineage-specific adaptations, it is also possible that such observations result from technical limitations in detecting highly divergent homologs or from gene loss events under neutral evolution. The distribution of OGs and PGs of SAHS among the species was visualized in a Venn diagram (Fig. 2D), which showed one PG for each of the species *R. varieornatus* and *H. exemplaris*. Two OGs were shared between *H. exemplaris* and *H. henanensis*, and one OG was shared between *R. varieornatus* and *P. metropolitanus*. Meanwhile, two SAHS-OGs were shared among all four tardigrade species. The maximum likelihood-based gene tree of SAHS (Fig. 2E) clustered *H. exemplaris* and *H. henanensis* together, and *R. varieornatus* and *P. metropolitanus* together. To provide a reference species phylogeny for comparison with gene-specific trees, we reconstructed a concatenated species tree (Fig. 2F) based on dataset of five conserved, single-copy orthologous proteins from the four tardigrade species and one outgroup species (*Caenorhabditis elegans*). The species tree provides a stable framework for interpreting the evolutionary history of SAHS family: the gene tree follows the species topology (concordant nodes), whereas some SAHS loci (orthologous clusters/networks) show discordant placements (Fig. 2A,C) consistent with gene duplication and lineage-specific evolution.

**Fig. 3:**
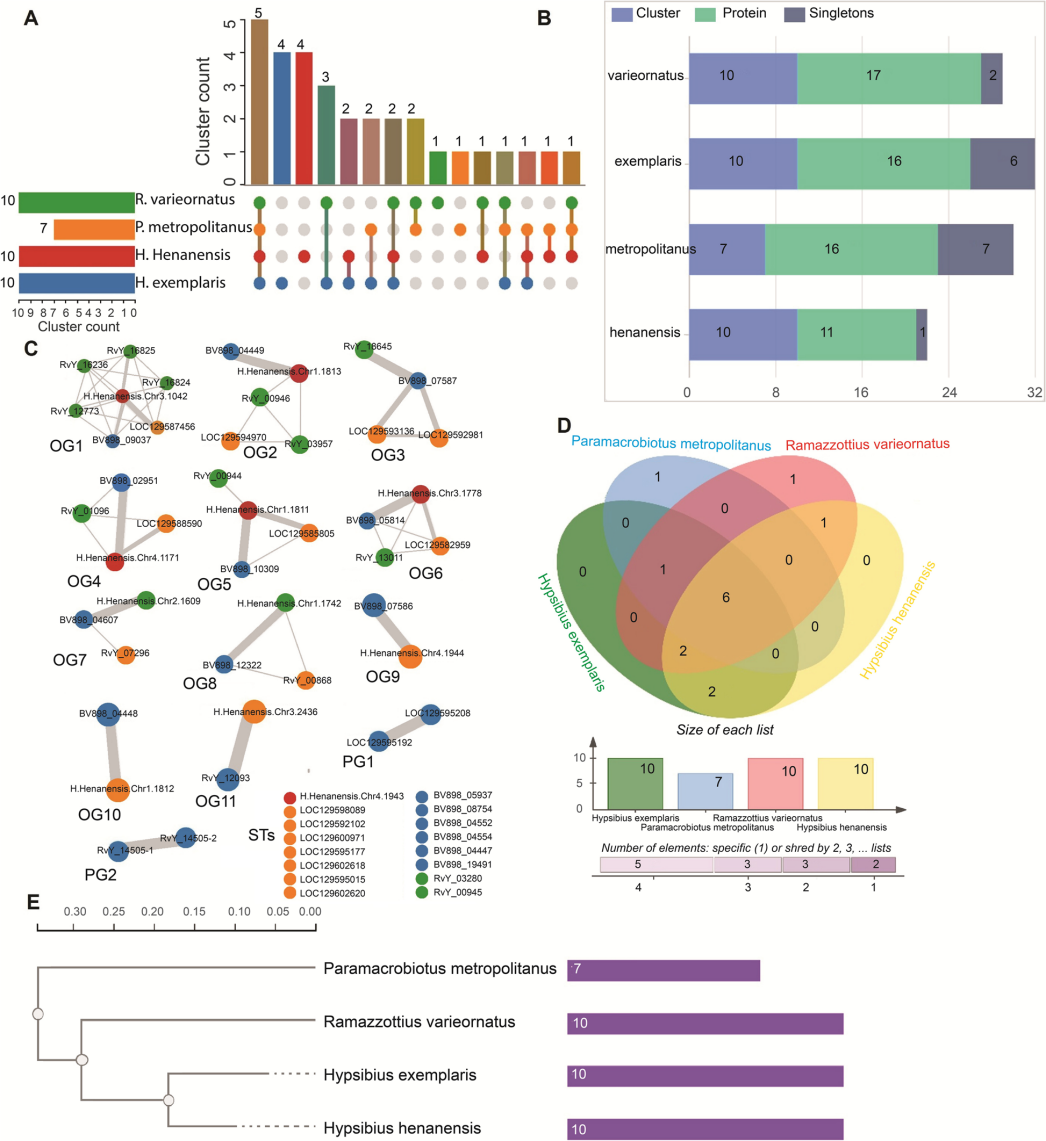
Analysis of orthologous clusters for CAHS proteins across the tardigrade species. **A** The UpSet plot illustrates the unique and shared orthologous clusters for CAHS proteins across species. The horizontal bar chart on the left depicts the count of orthologous clusters for each species, while the vertical bar chart on the right shows the number of clusters shared between species. The intersecting lines represent the overlapping sets. **B** Distribution of clusters, proteins, and singletons for CAHS proteins across the species. **C** Conserved ortholog groups (OGs), paralog groups (PGs), and singleton members (STs) of CAHS proteins in tardigrades. Edges widths represent numbers of interactions between the genes. **D** Venn diagram of cluster sharing for CAHS proteins across the species. **E** Gene tree of phylogenetic relationships based on the identification of highly conserved single-copy sequences of CAHS proteins across the tardigrade species. Locus tags correspond to: RvY, *R. varieornatus*; BV898, *H. exemplaris*, LOC, *P. metropolitanus*; and H.Henanensis.Chr, *H. henanensis*

The 60 proteins of CAHS were distributed into a total of 13 clusters and 16 STs (Fig. 3A-B). The clusters of CAHS were further differentiated into 11 conserved OGs and 2 PGs (Fig. 3C). Among the OGs, 3 were single-copy clusters representing a unique copy of the protein from each species. The STs of CAHS accounted for 26.67% of the total proteins. In species-wise distribution of STs, *P. metropolitanus* showed 7, *H. exemplaris* showed 5, *R. varieornatus* showed 2, and *H. henanensis* showed 1. The Venn diagram visualized the distribution of OGs and PGs of CAHS among the species (Fig. 3D). One PG was observed for each of the species *R. varieornatus* and *P. metropolitanus*. Two OGs were shared between *H. exemplaris* and *H. henanensis*, and one between *H. henanensis* and *R. varieornatus*. One OG was shared among *R. varieornatus*, *H. exemplaris*, and *P. metropolitanus*, and two among *H. henanensis*, *H. exemplaris*, and *P. metropolitanus*. Meanwhile, 6 of the CAHS-OGs were shared among all four species. The gene tree of CAHS (Fig. 3E) clustered *H. exemplaris* and *H. henanensis* together, while *P. metropolitanus* was clustered at the root. Similar to SAHS family, the gene tree of CAHS family shows concordance with species topology, whereas some CAHS orthologous clusters/networks show discordant placements (Fig. 3A,C)

### Molecular phylogenetic analysis, conserved sequence motifs and 3 d structural modeling of stress-response proteins

**Fig. 4:**
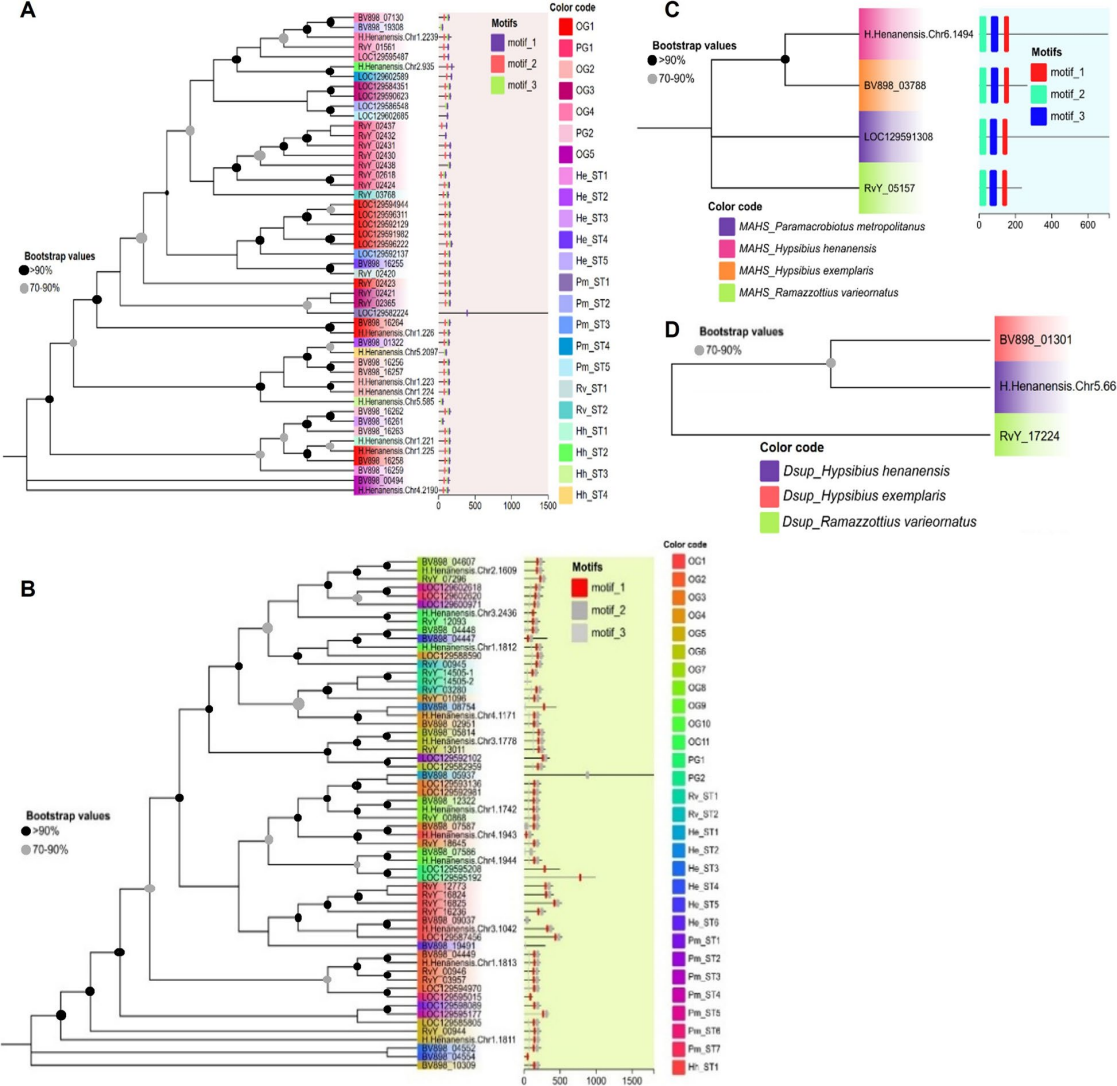
Maximum likelihood based molecular phylogenetic analysis of stress-response proteins in tardigrades. **A** Phylogenetic tree of SAHS. **B** Phylogenetic tree of CAHS. **C** Phylogenetic tree of MAHS. **D** Phylogeny of Dsup showing the phylogenetic relationships of newly identified Dsup (H.Henanensis.Chr5.66) during present study with other two other known Dsup proteins. Locus tags correspond to: RvY, *R. varieornatus*; BV898, *H. exemplaris*, LOC, *P. metropolitanus*; and H.Henanensis.Chr, *H. henanensis. *The color codes correspond to sequences from specific orthologous groups (OGs), paralogous groups (PGs), and singletons (STs). The locations of conserved motifs are shown in the middle panel, with each motif represented by a distinct color along the protein lengths (scaled at the bottom)

The evolutionary relationships within the SAHS, CAHS, MAHS, and Dsup protein families were examined using molecular phylogenetic trees based on the Maximum Likelihood algorithm (Fig. 4). The phylogenetic analysis of SAHS proteins in tardigrades (Fig. 4A) revealed clustering patterns similar to those observed in the orthologous analysis. Conserved motifs were mapped for all proteins, with *H. henanensis* (*Chr4.2190*) and *BV898_00494* forming the root cluster of the tree. A similar clustering pattern was observed for CAHS proteins (Fig. 4B), where proteins from *H. exemplaris* and *H. henanensis* formed the root clusters. Conserved motifs were also mapped across all CAHS proteins. For MAHS phylogeny (Fig. 4C), only one protein was identified from each species. The resulting phylogenetic tree clustered *H. exemplaris* and *H. henanensis* together, indicating their close evolutionary relationship. In contrast, the Dsup protein was found in only three of the four species (Fig. 4D) and was markedly absent in *P. metropolitanus*. Notably, in this study we identified the third Dsup-member (H.Henanensis.Chr5.66) in *H. henanensis* (Fig. 4D). The first Dsup-member (UniProt ID: P0DOW4) was identified in in *R. varieornatus* in 2016 (Hashimoto et al. 2016).

**Fig. 5:**
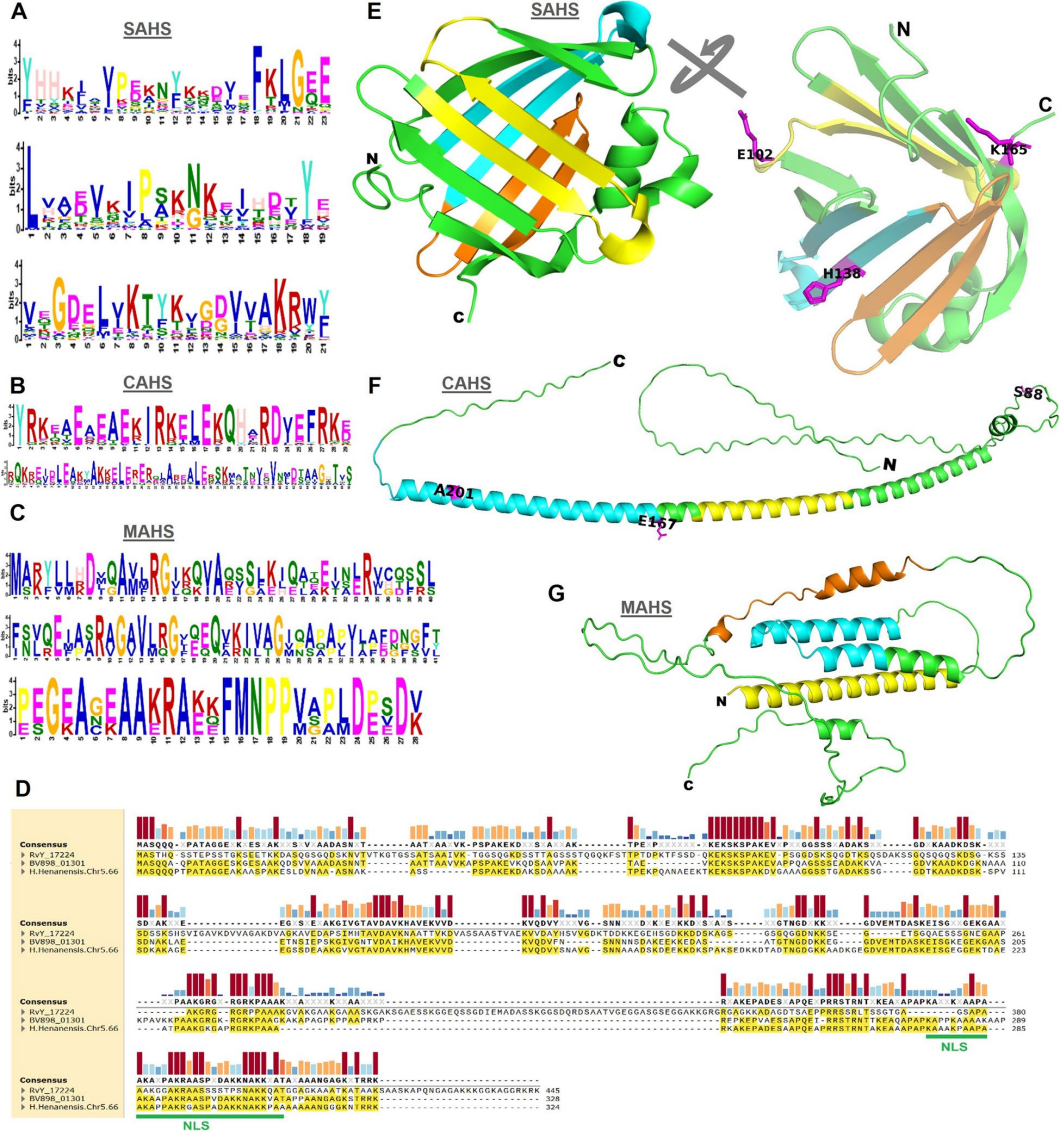
Conserved sequence motif analysis and molecular modelling of protein structure. **A** Conserved motif logo for SAHS. **B** Conserved motif logo for CAHS. **C** Conserved motif logo for MAHS. **D** MAFFT based multiple sequence alignment of Dsup showing some conserved short motifs among the three species. Lengths of small bars on the top represent the conserved residues in alignment. NLS, nuclear localization signal. **E** The crystal structure of SAHS1 under the PDB accession 5XN9 for *Ramazzottius varieornatus*. **F** Protein 3D structure predicted for CAHS1 from *R. varieornatus*. **G** Protein 3D structure predicted for MAHS from *R. varieornatus*. Color codes: yellow, first conserved motif; cyan, second conserved motif; orange, third conserved motif; magenta, candidate convergent sites

Conserved sequence motifs were identified within the SAHS, CAHS, and MAHS protein families, providing insights into their functional roles in stress tolerance (Fig. 5, and S1 Additional file). Three conserved motifs were identified in the SAHS family (Fig. 5A), while two motifs were detected in the CAHS family (Fig. 5C). The MAHS family exhibited three conserved motifs, which were highly consistent across all tardigrade species (Fig. 5E). Multiple sequence alignment of the Dsup protein using MAFFT revealed several conserved short motifs shared among the three species where it was present (Fig. 5G). The identification of these conserved motifs highlights their potential importance in maintaining protein stability and function under stress conditions.

**Fig. 6:**
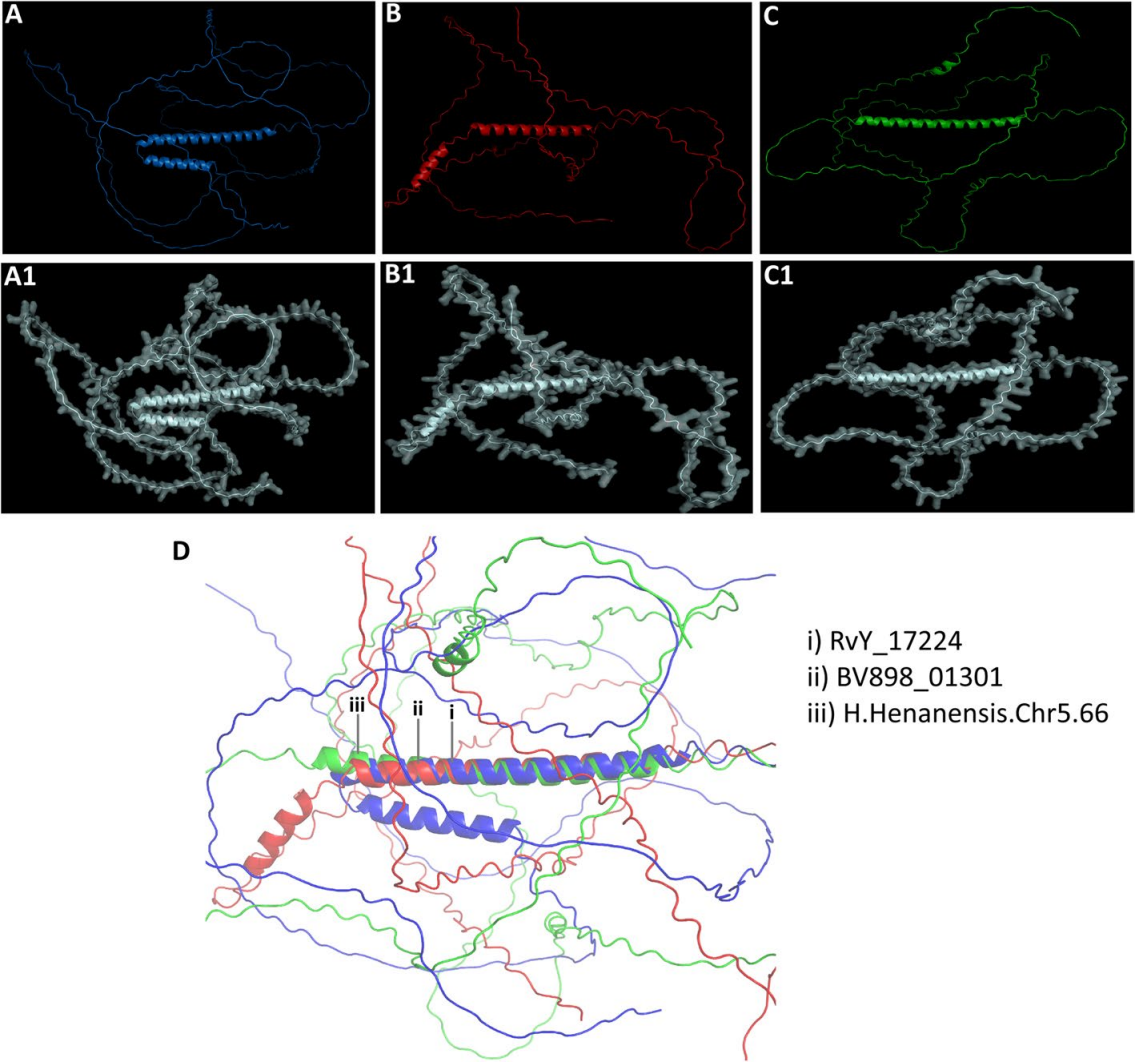
Molecular modeling of the damage suppressor protein (Dsup) across different tardigrade species. **A** Ribbon diagram and (**A1**) surface model of the Dsup (RvY_17224) in *Ramazzottius varieornatus*. **B** Ribbon diagram and (**B1**) surface model of the Dsup (BV898_01301) in *Hypsibius exemplaris*. **C** Ribbon diagram and (**C1**) surface model of the Dsup (H.Henanensis.Chr5.66) in *Hypsibius henanensis*. **D** Structural alignment of Dsup proteins across the three tardigrade species, highlighting the conserved α-helix regions

To further investigate the functional significance of these motifs, 3D structural modeling was performed for SAHS1, CAHS1, and MAHS proteins, using *R. varieornatus* as the model species (Fig. 5B, D, F). Notably, the 3D structural model of SAHS1 revealed the presence of β-sheet regions, which are absent in most other stress-response proteins. In contrast, the majority of these proteins exhibit a high degree of intrinsic disorder (Figs. 5, 6). These findings suggest that the structural properties of SAHS1 may play a unique role in stress adaptation mechanisms in tardigrades.

### Candidate convergent sites in TDPs

ConDor analysis of the SAHS family (S1 Additional file: Table S1) identified three alignment columns with a statistically significant excess of independent emergence events that map to residues E102, H138, and K165 in the reference SAHS1 sequence (UniProt J7MFT5). The column corresponding to K165 exhibited ten independent emergences to lysine (K) across the tree (present in 17 extant sequences) and was highly significant versus the simulated null (FDR = 0.00295), consistent with repeated parallel substitution to the same residue. The site mapping to E102 showed seven emergences dominated by glutamate (E; 15 sequences overall; FDR = 0.016), indicating a statistically supported excess of independent substitutions to an acidic residue. The column corresponding to H138 displayed seven emergence events involving multiple residue states (H/K/N/S) and was likewise significant (FDR = 0.0187), a pattern consistent with recurrent substitutions toward related biochemical classes rather than a single identical replacement. Importantly, when projected onto the SAHS structural model (Fig. 5B) these candidate sites localize within conserved sequence motifs identified by our motif-discovery analysis, providing mechanistic plausibility for their recurrent modification. Similarly, ConDor detected three CAHS alignment columns with significant excesses of independent emergences (S1 Additional file: Table S2) that map to S88, E167 and A201 in the reference CAHS1 sequence (UniProt J7M799). The site corresponding to A201 exhibited seven independent emergences (predominantly histidine substitutions) and was classified as convergent (FDR = 0.00215). The position corresponding to E167 showed eleven emergences, dominated by glutamate (E:10), and was also significant (FDR = 0.00215). The site mapping to S88 displayed eleven emergences, primarily serine with a minority of threonine substitutions (FDR = 0.00903). In all three cases the observed counts of emergence events substantially exceeded expectations under the simulated neutral model. When these candidate sites were projected onto the CAHS structural model (Fig. 5D), they localized to the conserved sequence motifs predicted in our motif analysis, supporting the interpretation that recurrent substitutions occur within functionally constrained regions.

### Comparative structural and molecular analysis of the dsup protein family

The Dsup protein family is well-documented for its crucial role in protecting DNA from radiation-induced damage, particularly in tardigrades. Despite its significance, the primary structure of Dsup proteins exhibits considerable variability across different tardigrade species, warranting a closer examination of its evolutionary adaptations. A BLAST search against the NCBI non-redundant database revealed no closely related proteins, indicating that Dsup represents a unique protein family. A comprehensive search for potential Dsup homologs across tardigrade species identified Dsup-like proteins in three of the four species examined (Figs. 4D, 5G). In this study, we identified a third Dsup member (H.Henanensis.Chr5.66) in *H. henanensis*, consisting of 324 amino acids. This is slightly shorter than the other two Dsup members, which contain 445 residues (for *R. varieornatus*) and 328 residues (for *H. exemplaris*). Pairwise sequence comparison for identity/similarity were conducted using BLOSUM62 algorithm as implemented in BioEdit software. Pairwise comparisons of this newly identified Dsup from *H. henanensis* revealed a similarity(identity) index of 0.6(0.54) with *H. exemplaris* and 0.32(0.23) with *R. varieornatus*. In comparison, the index between the other two Dsup members, from *H. exemplaris* and *R. varieornatus*, was 0.31(0.23). These results suggest that the newly identified Dsup from H. *henanensis* shares a greater similarity with *H. exemplaris* than with *R. varieornatus* (Fig. 4D). Additionally, we analyzed the subcellular localization of these Dsup proteins using the WoLF PSORT tool (https://wolfpsort.hgc.jp/), which predicted nuclear localization for all three Dsup proteins. A putative nuclear localization signal was also predicted near the C-terminus by the cNLS Mapper tool (https://nls-mapper.iab.keio.ac.jp/cgi-bin/NLS_Mapper_form.cgi) (Fig. 5G). These results suggest the H.Henanensis.Chr5.66 is a potential Dsup orthologue in *H. henanensis*.

Although overall sequence similarity among the three Dsup proteins is relatively low, certain motifs were strictly conserved among these proteins (Fig. 5G). These include the motifs KEKSKSPAKEVXPXXGGSSSXADAKS (positions 90–114) and AKGRGXRGRKPAAAK (positions 262–274), which are likely crucial for the protein’s function. These conserved motifs may play an essential role in the protein’s ability to protect DNA and maintain cellular integrity during extreme stress conditions. The divergence in Dsup protein sequences among the three species suggests that the primary structure of Dsup has been under weak selective pressure during evolution, possibly due to the intrinsically disordered nature of the protein. Similar sequence diversifications have been observed in other intrinsically disordered proteins, such as late embryogenesis abundant (LEA) proteins, which function as desiccation protectants. The flexible structure of these proteins may serve as a physical shield for DNA or other cellular components during desiccation or other forms of stress, providing an important adaptation to extreme environments.

**Fig. 7:**
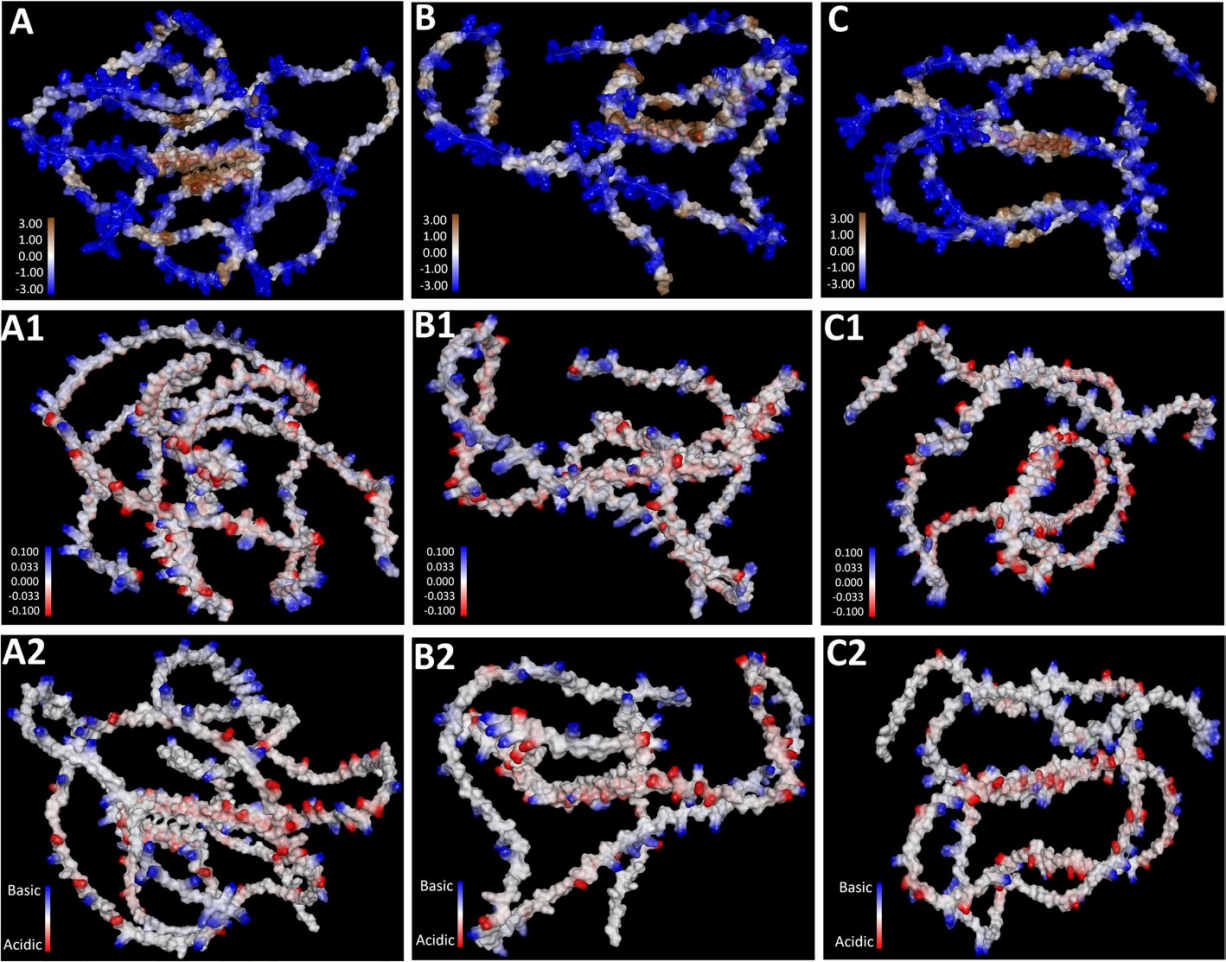
Comparative analysis of molecular attributes of Dsup across three tardigrade species. Hydrophobicity distribution (blue indicating hydrophilic to brown indicating hydrophobic) of Dsup in *Ramazzottius varieornatus* (**A**), *Hypsibius exemplaris* (**B**), and *H. henanensis* (**C**). Charge distribution of Dsup in *R. varieornatus* (**A1**), *H. exemplaris* (**B1**), and *H. henanensis* (**C1**). Ionizability distribution (from blue for basic to red for acidic) of Dsup in *R. varieornatus* (**A2**), *H. exemplaris* (**B2**), and *H. henanensis* (**C2**)

Interestingly, despite the sequence variability, structural analyses revealed notable similarities among Dsup proteins of the three tardigrade species (Figs. 6, 7). Molecular modeling of the three Dsup proteins was conducted to compare their structural features (Fig. 6A-C, ribbon models; Fig. 6A1-C1, surface models). Structural alignment of these proteins (Fig. 6D) demonstrated that Dsup proteins from *R. varieornatus*, *H. exemplaris*, and *H. henanensis* share a conserved α-helix region. Pairwise structural superposition of predicted Dsup homologs revealed a conserved core with RMSD values of 3.79 Å (RvY_17224 vs BV898_01301), 3.91 Å (H.Henanensis.Chr5.66 vs BV898_01301), and 4.80 Å (H.Henanensis.Chr5.66 vs RvY_17224). These RMSD values (≈3.8–4.8 Å) indicate moderate fold conservation—consistent with a shared structural element (the α-helical core) that persists across Dsup homologs despite sequence-level divergence. This α-helical core may contribute to protein stabilization or other important function such as DNA protection under extreme conditions. Functional studies are required to decipher the role of this conserved structural motif across the Dsup proteins.

Further comparative analyses of hydrophobicity, charge distribution, and ionizability revealed highly similar protein behavior across the three species (Fig. 7). Notably, all three proteins exhibited a broad hydrophobic region in the central portion (Fig. 7A-C) and a positively charged C-terminal domain (Fig. 7A1-C1). This charge distribution pattern is highly conserved, suggesting that these structural features are essential for maintaining protein stability and function during stress exposure. The charge (Fig. 7A1-C1) and ionizability distributions (Fig. 7A2-C2) suggested that the electrostatic properties of Dsup may play a crucial role in stabilizing DNA under stress conditions. These findings suggest that Dsup functions in a flexible rather than rigid conformation, potentially acting as a molecular shield to protect DNA from damage during environmental stress. The structural conservation of α-helical regions and charge distributions underscores the functional importance of Dsup in stress resistance, supporting the hypothesis that this protein plays a universal role in DNA protection across tardigrade species.

The integrative bioinformatics approach used in this study has provided compelling evidence for the patterns consistent with convergence evolution of stress-response proteins in tardigrades. Despite their phylogenetic distance, the SAHS, CAHS, MAHS, and Dsup families exhibit shared structural and functional features that enable tardigrades to thrive in extreme environments. While intrinsically disordered regions are common in many proteins, the specific conserved motifs and structural features of the SAHS, CAHS, MAHS, and Dsup families are absent in other taxa, making these families unique to tardigrades. This pattern, along with BLAST analysis and phylogenetic clustering, distinguishes these proteins from related families and supports the hypothesis of convergent evolution in response to extreme environmental pressures. Nonetheless, we acknowledge that further functional assays and comparative evolutionary analyses are needed to definitively establish convergent evolution. These findings highlight the importance of conserved protein motifs and structural features that contribute to the unique stress tolerance mechanisms in tardigrades. The identification of these molecular adaptations offers new insights into the evolutionary strategies of extremophiles and underscores the potential applications of tardigrade stress-response proteins in biotechnology and other applied sciences.

## Discussion

The extraordinary resilience of tardigrades to extreme environmental conditions is largely attributed to their unique stress-response proteins, which play a crucial role in enabling survival under desiccation, high radiation, and extreme temperatures (Hengherr et al. 2009; Jönsson et al. 2008). In this study we employed an integrative bioinformatics approach to investigate the genetic, structural, and evolutionary characteristics of the SAHS, CAHS, MAHS, and Dsup protein families in tardigrades, providing insights into their evolutionary conservation and functional diversity. Our findings indicate that both stabilizing and diversifying selection have shaped these protein families, with conserved motifs and structural adaptations underpinning their essential roles in stress tolerance.

We found the variation in gene copy numbers and exon-intron structures across species, which reflect species-specific adaptations to different environmental pressures. SAHS and CAHS genes displayed expansion in *R. varieornatus* and *H. exemplaris*, suggesting that gene duplication may have conferred an adaptive advantage by increasing stress tolerance (Ebadi et al. 2023; Kondrashov 2012; Magadum et al. 2013; Qian and Zhang 2014; Shen et al. 2025). Conversely, the MAHS gene family exhibited strict conservation across all species analyzed, highlighting its essential and possibly irreplaceable role in mitochondrial protection under extreme conditions (Rolsma et al. 2024; Tanaka et al. 2015). The observed variation in exon and intron sizes may be associated with regulatory and functional adaptations that contribute to stress tolerance; however, we acknowledge that these patterns might also arise under neutral evolutionary processes, and further experimental validation is needed to disentangle adaptive from non-adaptive changes. Although the differences in gene copy numbers and sequence conservation among species could reflect adaptive responses to environmental challenges, we cannot rule out that some of these differences may also result from neutral evolutionary processes such as genetic drift. The absence of Dsup homologs in *P. metropolitanus* raises important questions about alternative stress-protective mechanisms in this species, suggesting that different tardigrade lineages may have evolved distinct molecular strategies to achieve genomic stability. Despite species-specific variations, our orthologous clustering analysis showed significant evolutionary conservation in stress-response proteins, supporting the hypothesis that tardigrades have developed shared molecular strategies to counteract environmental stressors (Møbjerg and Neves 2021). The candidate convergent sites in TDPs show a statistically significant excess of independent emergence events. The recurrence of substitutions—including repeated replacement by chemically similar residues (e.g., serine/threonine at S88, glutamate at E167, and lysine at K165) or by residues that alter local charge or polarity (e.g., histidine substitutions at A201)—is notable because all three SAHS and CAHS candidate sites localize to the conserved sequence motifs we identified and to structural features in our models. Localization within conserved motifs increases the biological plausibility that these changes are functionally relevant rather than stochastic, and the pattern of residue replacement (identical in some cases, functionally similar in others) suggests selection for particular biochemical properties at these positions rather than neutral drift.

Our analysis also identified a novel Dsup homolog (H.Henanensis.Chr5.66) in *H. henanensis*, expanding the known diversity of this protein family and suggesting lineage-specific divergence in DNA protection mechanisms. Structural modeling confirmed that Dsup proteins across species share a conserved α-helical region, essential for protein stabilization and chromatin interaction (Mikhail Zarubin et al. 2024a, b). To our knowledge, prior studies have not directly compared structural models of Dsup homologs across multiple tardigrade species to highlight conserved α-helical features. While the presence of intrinsically disordered regions is not unique, the structural conservation of this specific region adds a layer of insight to the hypothesis that Dsup proteins may have evolved a shared structural element contributing to DNA protection. However, additional experimental validation is necessary and functional studies are required to decipher the role of this conserved structural motif. The positively charged C-terminal regions in all three Dsup homologs further support electrostatic interactions with nucleosomal DNA, aligning with their proposed role in chromatin stabilization and genome integrity maintenance (Mínguez-Toral et al. 2020; Møbjerg and Neves 2021; Zarubin et al. 2023, 2022). Additionally, nuclear localization predictions confirmed that Dsup proteins primarily function within the nucleus (Adam et al. 1989; Aguilar et al. 2023), further reinforcing their role in DNA protection from radiation-induced damage (Kasianchuk et al. 2023). These findings suggest that Dsup-mediated DNA stabilization is a universal mechanism among tardigrades possessing this protein, while species lacking it may have developed alternative protective adaptations.

The broader implications of these findings extend beyond evolutionary biology, as they provide a framework for potential biotechnological applications of tardigrade stress-response proteins. Given their exceptional protective properties, Dsup proteins could be leveraged for biomedical, agriculture, and space-related applications, including radiation protection in human cells and genetic engineering for enhanced stress tolerance **(**Del Casino et al. 2024**; **Westover et al. 2020; Ye et al. 2023**)**. The stabilizing properties of CAHS and MAHS proteins further suggest potential applications in biopreservation, pharmaceuticals, and synthetic biology, where their ability to prevent protein aggregation and enhance cellular stability could be beneficial (Jönsson 2019; Piszkiewicz et al. 2019; Sanchez-Martinez et al. 2023; Schill et al. 2009). Future research should focus on functional validation of these proteins in heterologous systems, particularly in radioprotective applications and biological storage solutions.

A key limitation of our study is that all functional inferences are computational and therefore necessarily provisional. Bioinformatic signals—conserved motifs, statistically supported excesses of independent substitutions, and co-localization of candidate sites on predicted structural models—are valuable for hypothesis generation but do not establish molecular mechanism or physiological effect. Further, computational models do not capture cellular context, expression dynamics, post-translational modifications, or biophysical behaviors that determine function in vivo. For these reasons we treat the sites and motifs reported here as prioritized candidates for experimental follow-up, which are necessary to confirm whether the recurrent substitutions we identify are adaptive and to elucidate their mechanistic roles in tardigrade stress tolerance.

## Conclusion

In conclusion, this study advances our understanding of the molecular adaptations underlying tardigrade stress tolerance, particularly through the conserved and species-specific evolutionary trajectories of the SAHS, CAHS, MAHS, and Dsup protein families. The identification of shared structural motifs and evolutionary conservation highlights the potential convergent signatures of stress-response proteins in tardigrades, reinforcing their role in maintaining cellular and genomic integrity under extreme conditions. Our integrative analysis provides a comparative framework for future functional validation studies.

## Materials and Methods

### Tardigrade species and protein families

In this study, we focused on four major stress-response protein families in tardigrades: SAHS (secretory abundant heat-soluble), CAHS (cytoplasmic abundant heat-soluble), MAHS (mitochondrial abundant heat-soluble), and Dsup (damage suppressor protein). The tardigrade species analyzed were *R. varieornatus*, *H. exemplaris*, *P. metropolitanus*, and *H. henanensis*, which were selected for their well-documented ability to withstand extreme environmental conditions, and the availability of their well annotated genomes. Complete, high-quality, and well-annotated genomic data are available only for these four species of tardigrades in public databases. To date, no study has conducted a comprehensive genome-wide and proteome-wide analysis using all four tardigrade species to identify and characterize orthologs in the stress-response protein families SAHS, CAHS, MAHS, and Dsup. The full extent of ortholog diversity in these protein families remains unclear. Therefore, this study aims to provide an in-depth, integrative bioinformatics analysis of stress-response proteins in tardigrades, utilizing genome-wide and proteome-wide approaches to elucidate their ortholog diversity, evolutionary relationships, and conserved functional and structural properties.

### Genome and proteome sequence retrieval and analysis

Genome and proteome sequences for the species *H. exemplaris* (genome assembly nHd_3.1 with accession ID GCA_002082055.1) and *P. metropolitanus* (genome assembly Prichtersi_v1.0 with accession ID GCA_019649055.1) were retrieved from Ensembl Metazoa database (https://metazoa.ensembl.org/index.html) using BioMart data mining tool. Genome assembly of *R. varieornatus* (GenBank assembly GCA_001949185.1) was retrieved from NCBI database. Genome assembly of *H. henanensis* (GWH accession number GWHDUDB00000000) was retrieved from The National Genomics Data Center (NGDC) database of China (https://ngdc.cncb.ac.cn/gwh/).

Representative query sequences/motifs (UniProt IDs: SAHS1, J7MFT5; CAHS1, J7M799; MAHS, A0A1D1V3Z0; and Dsup, P0DOW4) were BLAST searched locally (BLAST parameters: S1 Code). The sequences for BLAST hits (genes) were either retrieved using BioMart or extracted and assembled from the genome assembly using Python scripts (Supplementary files: S2 Code and S3 Code). Multi FASTA files of sequences were further refined for gene IDs in header line (IDs-simplified) using TBtools-II (Chen et al. 2023).

### Gene structure analysis

Gene structure analyses of the protein families were performed to determine exon and intron lengths across the different tardigrade species. The gene structures were annotated by identifying exon-intron boundaries using the GSDS 2.0 (Hu et al. 2015). The identified gene models were manually curated, and their lengths were compared across species. Differences in exon and intron lengths can indicate structural variation, while conserved exon-intron architectures across species may suggest functional or evolutionary constraints preserving gene structure.

### Orthologous cluster identification

Orthologous clustering of stress-response proteins across tardigrade species was performed using the *OrthoMCL* algorithm as implemented in OrthoVenn3 (Sun et al. 2023). Protein sequences of the specific protein family were used as input to generate orthologous groups (OGs). Specific parameters included substitution models JTT+CAT, E-value of 0.001, and inflation value 1.50. The resulting ortholog clusters were manually examined to assess shared and unique clusters across species. Single-copy clusters were identified by selecting those containing one orthologous gene per species. Overlap of orthologous clusters, and unique and shared groups were visualized with Venn diagrams and UpSet plots.

### Molecular phylogenetic analysis

For molecular phylogenetic analysis, multiple sequence alignment (MSA) was performed using algorithms either MUSCLE in MEGA11 (Tamura et al. 2021) or the *MAFFT* (Katoh et al. 2019). The MSA was visualized using software tools BioEdit (Hall 1999) and SnapGene (https://www.snapgene.com). Maximum likelihood algorithm based phylogenetic trees were constructed (Additional file S4 Code) using IQ-TREE2 (Minh et al. 2020). For each protein family we used ModelFinder (implemented in IQ-TREE2) to identify the best-fit substitution model based on information-theoretic criteria (AIC, AICc and BIC). The selected models were WAG+G4 for SAHS, Q.insect+F+G4 for CAHS & Dsup, and VT+F+G4 for MAHS. The trees were visualized using tvBOT server (Xie et al. 2023).

For a species reference tree, single-copy orthologous proteins (APC8, BCS1, CLP1, DNAligase, RRP7) for the four tardigrade species and an external outgroup were identified and extracted from OrthoDB server (Tegenfeldt et al. 2025). Protein sequences for each locus were aligned separately with MAFFT and trimmed with trimAl v1.5 (Capella-Gutiérrez et al. 2009). Trimmed per-gene alignments were concatenated into a partitioned supermatrix using a custom Python script; partition boundaries were retained for each gene. A partitioned maximum-likelihood species tree was inferred with IQ-TREE under the best-fit substitution model (LG+I+G4), and node support was assessed using bootstraps.

### Motif and structural analysis

Conserved sequence motifs within the protein sequences were identified using *MEME Suite* (https://meme-suite.org/meme/) with default settings to detect short, conserved motifs across multiple species. The motifs were also mapped onto the corresponding phylogenetic trees to assess their evolutionary conservation. While initial motif analysis of CAHS, and SAHS proteins was reported (Yamaguchi et al. 2012), we re‐examine these motifs using updated algorithms and expanded sequence datasets. By integrating motif identification with structural modeling and phylogeny, we provide a refined view of motif conservation and its structural implications across tardigrade species. This integrative approach not only validates previous findings but also uncovers additional subtle variations that may underlie species‐specific adaptations.

The crystal structure of SAHS1 was downloaded from the Protein Data Bank (PDB) under the accession 5XN9. For the other three protein families, for which experimental structures are not available, 3D structural models were predicted using the AlphaFold Server *(*Abramson et al. 2024*).* The models were visualized using PyMOL molecular graphics system v3.1.3 (Schrödinger, LLC), and Discovery studio visualizer version v24.1.0.23298 (BIOVIA, Dassault Systèmes, San Diego, USA). Protein structural alignment was performed in PyMOL. Hydrophobicity distribution, charge distribution, and ionizability of Dsup proteins were visualized using Discovery studio visualizer.

### Evolutionary convergence analysis of TDPs

Evolutionary convergence analysis was performed using the ConDor webserver (Morel et al. 2024). The ConDor was run in emergence mode with default simulation settings to generate null distributions of emergence counts and accepted sites as significant only after the server’s multiple-testing correction (Benjamini–Hochberg FDR; threshold FDR < 0.05). Alignment column indices from ConDor were mapped to reference residue numbers in the respective TDP. The candidate sites were also projected onto predicted structural models of TDPs.

## Supplementary Information

Additional file 1. S1. Code for local BLAST search

Additional file 2. S2 Code. Python script for extracting sequences

Additional file 3. S3 Code. Python script for combining extracted sequences into one multi-FASTA file

Additional file 4. S4 Code. IQ-TREE2 phylogeny code

Additional file 5. S1. Additional file. motif analysis

Additional file 6. S2. Additional file. ConDor analysis

## Data Availability

Data supporting the conclusions of this article are included within the article and its additional files. Accessions and links to data are provided in the methods section. Further inquiries can be directed to the corresponding author.

## Abbreviations

BLOSUM62: BLOcks SUbstitution Matrix 62
BLAST: Basic local alignment search tool
CAHS: Cytoplasmic abundant heat-soluble
Chr: Chromosome
DNA: Deoxyribonucleic acid
Dsup: Damage suppressor protein
LEA: Late embryogenesis abundant
MAFFT: Multiple alignment using fast fourier transform
MAHS: Mitochondrial abundant heat-soluble
MSA: Multiple sequence alignment
NLS: Nuclear Localization Signal
OGs: Orthologous groups
PGs: Paralogous groups
SAHS: Secretory abundant heat-soluble
TDPs: Tardigrade intrinsically disordered proteins
