# Supplementary material for: Integrative bioinformatics analysis of potential convergent evolution in tardigrade stress-response proteins
Source: Stress Biol. 2026 Mar 23;6(1):21. doi: 10.1007/s44154-026-00286-5 (PMC13006486; doi:10.1007/s44154-026-00286-5)
Supplement: Supplementary file 5 — Additional file 5. S1. Additional file. motif analysis [file 44154_2026_286_MOESM5_ESM.pdf]

Supplementary material: S1 Additional file

Motif Analysis_SAHS_Logo 1

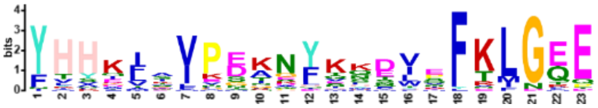

Name	Start	*p*-value	Sites		
LOC129595487	65	1.25e-23	INEFSASGDE	YHHKFSVPSKNYVQDLPFKLGEE	RQAAFNGTNY
LOC129596222	103	9.82e-23	WHKLWKEGDH	YHHRIKVPEKNYKLDVEFKLGEE	GTGSFNNTNF
LOC129591982	81	9.82e-23	WHKLWKEGDH	YHHRIKVPEKNYKLDVEFKLGEE	GTGSFNNTNF
RvY_02423	81	4.45e-22	FHKIWKEGDH	YHHQISVPDKNYKNDVNFKLNEE	GTTQHNNTEI
H.Henanensis.Chr1.225	80	1.80e-21	IHTIYKQGDH	YHHVFAIPEKNFQKDIEFTLGQE	SSVKHGEHEV
BV898_16262	78	1.99e-20	IQKIYKQGDH	YHHIFALPDKNFEKDIEFTLGQE	VEIKQGEHIA
BV898_16264	80	2.28e-20	VHKLWKEGDH	YHHQIIIADKSYKQDIQFKLGEE	GRTAHNGTEV
RvY_02430	82	5.01e-20	HLNHYKKGDD	YHHKIIVKEAEYKNDVVFKLGQE	SAGSYNGSSF
RvY_02437	32	1.56e-19	WVNTYKKGDE	YHHKIIIKEAGYTNDVVFKLGQE	SAGSHNGSSF
RvY_02431	83	2.00e-19	WIHHYKKGDE	YHHKIIINDAHYKNDIVFKLGQE	SAGSYNGSSF
RvY_01561	61	2.26e-19	YNEFTQNGDE	YVHKFSVPSANYHQELPFKLGEQ	RTGQYRGKDF
H.Henanensis.Chr1.221	81	2.55e-19	IHTIYKQGDH	YHHIFAVPDKNLQRDTEFTLGQE	FVKHGEQEVK
LOC129592137	77	1.32e-18	THELWKEGDK	FHHKIAAPEVNYKKHICFKLGEE	GKSSYNGTEF
BV898_16263	78	1.48e-18	IHSFYKQGEH	YHHILSLPDKNINKDIEFTLGQE	VEIKHGEHSL
BV898_16258	78	4.06e-18	VHTFYKQGDH	YHHIFAIPDKNFEKNIEFNLGAE	SSAKHGEHEV
RvY_02424	72	5.05e-18	TLHTFKKGDE	YHHKIVVKEAGYINDVIFRLGQE	TPGSYNGQQF
RvY_02618	23	4.59e-17	TLQTFKKGDK	YHHKIVVEEAGYINDVIFRLGRE	TPGSYNGQQI
LOC129592129	77	5.62e-17	YHKFWKEDDH	FHHGIAVTEKDFKKFVEFKLGEE	GTLTWNNTEF
BV898_16259	73	1.51e-16	IHTIYKQGDK	YHHEVSIPSKNFKKAIEYTLGTE	TDVQHGPHTI
H.Henanensis.Chr1.224	69	2.71e-16	SIVISRSGDQ	YTSELQVPEHNYVSSWSFKMGEE	GTKVEPKFGN
H.Henanensis.Chr1.223	69	2.71e-16	IIVLSRSGDQ	YTSELQVPEHNYVSSWSFKMGEE	GTKVEPKNRN
LOC129594944	81	4.35e-16	YHKLWKEGDH	FHHGLAVPDKQLKKFVQFKFGEE	QSLTFNNTEF
LOC129596311	82	5.77e-16	YRHLWKEGGQ	FHHAVAVPERNFQAVFQFTLGQE	ATLNVNGTEY
BV898_16255	81	1.10e-15	FQKFWQDGEH	FHHKITVPTKNYTLQHKFTLGQP	GKATFNNVEF
H.Henanensis.Chr1.226	69	2.99e-15	EGPLAAGYGG	NHKTIIIKDKAYKQDIQFKLGEE	GKTSHNGTEV
H.Henanensis.Chr1.2239	67	5.08e-15	NEFVDNGNGE	YIYKFNVENTNYHQELPFKLGEQ	RKSAYNGTEF
BV898_16257	66	7.84e-15	TLSITREGET	YTSKLEVPSNNFSSTWTFKIGEE	GTKVEPKFEN
BV898_16256	66	3.59e-14	TLSITREGET	YTNKLEVPSNNFSSTYSFKIGEE	GTKVEPKFEN
RvY_02365	84	4.60e-14	KYWKEAGEDH	YHVQTSFPGTEHKMETSFKMGQE	GTLSHDGVDL
H.Henanensis.Chr2.935	108	4.60e-14	LRSADGKVGS	YVHKFGIPGAKYNHTVPFVLDEE	KTVVTNATSL
BV898_07130	67	4.60e-14	NEFLDQGNGE	YLYKFRVENAAYKQELPFKLGET	RKSTYNGTEF
RvY_02420	77	6.38e-14	FIKFWKEDDH	FHLKVTVPEISYKMELEFTMGLQ	SKSTFNGTSF
LOC129602589	101	1.12e-13	HEFSRINSTT	YAHAFWIVDTPYKQNLPFELGRE	YPHSYNGTAF
RvY_02421	83	1.32e-13	ITIYKEGDSH	YHAQMTFPGTDHKKEWDFKIGQE	GTYSMDGTEV
H.Henanensis.Chr4.2190	55	2.55e-12	VARIWTDGEY	IHYDASIPSQNFMHNVAFKLGVD	TELNHGKNRI
LOC129584351	81	6.49e-12	THEYIEEGEQ	FLHKVKIVDGGQEYEVRFKLNEE	ATFHLGNEPD
BV898_00494	65	6.49e-12	VLKLWCDGED	FHYDAGILEAKFKHSVTFKLGTP	TELNHGNKIV
RvY_03768	56	6.97e-12	FLTYRREGDD	YYAETFVPGITFTQSLPFHFGQE	GSGKRFGTSM
LOC129590623	85	7.48e-12	HDFVRKSDDD	YVHKVKIGQGEDQYQVSFKLNEE	GTLHKAGAPE
BV898_01322	69	2.26e-11	ITITRDDGDN	YKVLLDVPAINFTSTWNLRLGEE	MVMDEFGSGM

Motif Analysis_SAHS_Logo 2

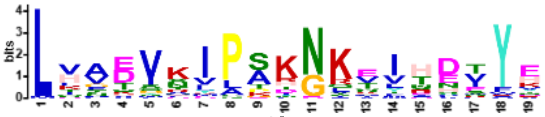

Name	Start	*p*-value	Sites		
H.Henanensis.Chr1.225	123	3.44e-20	KIKYSEEGDK	LVADVSIPSKNKNIHDVYE	VQGEELVKTY
H.Henanensis.Chr1.224	115	3.44e-20	KFTFTEDGDK	LLATVKIPSKNKEIHDVYE	VTGDELVKTC
BV898_16259	116	1.40e-19	KLKYTEDGEK	LVADVQIPSKNKQIHDIYE	VQGDTLTKTY
BV898_16258	121	1.76e-17	KIKYAEDGDK	LVADVRIAAKNKHIHDVYE	VQGEELVKTY
LOC129594944	124	7.33e-17	KITYSEKDKD	LHVDVKIPAKGVTIHDVYH	VEGEELVKSY
BV898_16263	121	8.41e-17	KIKYFEDGNK	LVADVSIPAKGKSIHDVYD	VQGDQLIKSY
H.Henanensis.Chr1.221	123	5.97e-16	KVKYSEDGDK	LVTDVSIPAKNKNIHFVYE	VQGEELIKTY
BV898_16261	30	1.79e-15	KTKYSEDGEK	LVADVSIPTKGKTIRSEYE	VQGDQLIKTY
BV898_16262	121	1.79e-15	KTKYSEDGEK	LVADVSIPTKGKTIRSEYE	VQGDQLIKTY
RvY_02423	124	3.23e-15	KYKYTEDGGN	LKAEVHVPSRNKVIHDEYK	VNGDELEKTY
BV898_16264	123	3.23e-15	TFKYTEVGDN	LQNEVKIPSKNKTISDSYV	VKGDELEKTY
RvY_02618	66	3.63e-15	TVNYEEQGGA	LVGTVKYPAHNKVIHNTYE	MDGQNLAKTS
H.Henanensis.Chr1.223	115	4.07e-15	KFIFTEDGDK	LLATVKIPSKNKEIDYTYV	VTGNELTKTY
LOC129592129	120	8.04e-15	KYKYTEENKD	LHVEVKVPAKDKVVHDTYH	VEGEQLVKTY
BV898_16257	112	8.99e-15	KYTFTEEGEK	LLVTVKIPARGKEVTEVYE	VTGDELVKTY
BV898_16256	112	8.99e-15	KYTFTEEGEK	LLVTVKIPARGKEVTEVYE	VTGDELVKTY
LOC129596222	146	6.82e-14	KYKYTEEGKD	LHVEVNLLTHNKVINDIYH	VDGDELVKTY
RvY_02424	115	7.55e-14	TVNYEEQGGA	LVGTIKYPAHNKVIHNTYK	MDGQNLTKTS
BV898_16255	124	7.55e-14	KYLYAELGND	LHVEITVPSKNKTVSDSYH	VFQNGTELEK
H.Henanensis.Chr1.226	112	1.02e-13	SFKYTEDGDN	LNNEVKIASKNKVITDSYV	VKGDELEKTY
LOC129591982	124	1.68e-13	KYKYTEEGKD	LHAEINLLTHNKVITDLYH	VEGDELVKTY
LOC129584351	126	7.08e-13	YVYKEEGPNK	LTVHMKIPSKNKELKECYN	VEGDKIVKNY
LOC129596311	125	5.51e-12	RFTYTEENGD	LHATVNVPKKNSKFHDKYH	VVGDELVKTF
RvY_02420	120	7.11e-12	TYTFSEDGDI	LKSENFIPSRNLTIFDTYR	VNGTEMEVHS
BV898_19308	23	7.43e-11	LYKFTVDGEL	LKFESKILPDGREVTHTYY	SNADGFVKQF
BV898_07130	110	7.43e-11	SYKFTVDGEL	LKFESKILPDGREVTHTYY	PNADGFVKQF
BV898_01322	112	8.02e-11	RYNFTEDGDK	LQAHVKISAIGKQYNENYE	VVGQELIITY
RvY_02421	126	8.66e-11	KYVYTENGDQ	LDSKLNIPSKNTEMTHTYK	VTGDELEHIF
LOC129592137	121	1.27e-10	FKYTEQASTD	LTMDVTMPGYNKTIVDIWH	VDGDELVKTF
H.Henanensis.Chr4.2190	98	1.71e-10	IIRYTEEDGK	LIADGDIPATGQHLHNIFD	AQGDELIKIL
H.Henanensis.Chr5.585	28	2.48e-10	YFKFTEIGEK	LHADIVEPKINRMYEEVYE	IKGDEMIKTI
RvY_02365	127	2.87e-10	KYVCTEDGEQ	LITKINIPSKNQETIVTYT	ATGDDLEQTF
H.Henanensis.Chr1.2239	110	4.44e-10	SYKFTVDGDK	LTFESKILPDGRDVTHTYT	PENGGFVKQF
LOC129590623	130	7.83e-10	FVYKETSGNK	LVVQQRVASKNIVLEEIYN	VQGDKIMKEY
BV898_00494	107	9.66e-10	VITYTEEDGK	LIADGVIAAKNLILHNVFA	AQGDVLIKTY
RvY_02438	128	4.86e-9	SVKYDEKDDA	LVGQVMLPSNNATYKNEFK	VEGDFLVKTS
RvY_03768	99	2.65e-8	KYTFREKGPE	MHCEVELPELNQKFNEWCK	VEDGELVKVF
H.Henanensis.Chr5.2097	75	5.44e-8	SHVFTKDGDK	LVGAYTRNSTGKAWTETYE	VKGKEMIKTF
LOC129586548	92	4.34e-7	ACTFSLDGNK	LHQVQRDPKTNLETTAERE	FTGDELITTL

Motif Analysis_SAHS_Logo 3

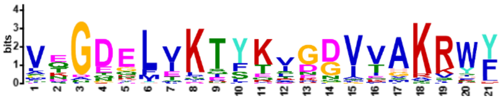

Name	Start	*p*-value	Sites
H.Henanensis.Chr1.225	142	1.87e-23	KNKNIHDVYE VQGEELVKTYKVGDVVAKRWF KKIANA
BV898_16258	140	1.87e-23	KNKHIHDVYE VQGEELVKTYKVGDVVAKRWF KKIAQ
LOC129591982	143	2.70e-23	HNKVITDLYH VEGDELVKTYKVGDVQAKRWY KRKSHDQATT
RvY_02423	143	5.59e-23	RNKVIHDEYK VNGDELEKTYKVGDVTAKRWY KKSSSS
BV898_16264	142	4.12e-22	KNKTISDSYV VKGDELEKTYKINDVVAKRWY KKHAHEPSTA
LOC129596222	165	6.57e-22	HNKVINDIYH VDGDELVKTYKSGDVQAKRWY RRKSHDQATT
H.Henanensis.Chr1.226	131	1.20e-21	KNKVITDSYV VKGDELEKTYKINDVTAKRWY KKHVQQASTA
H.Henanensis.Chr1.221	142	1.61e-21	KNKNIHFVYE VQGEELIKTYKVGDVVGKRWF KKIANA
BV898_16259	135	1.61e-21	KNKQIHDIYE VQGDTLTKTYKVGDVVAKRWF TREANPTA
BV898_16263	140	3.25e-20	KGKSIHDVYD VQGDQLIKSYKVGDVVAKKWF KKVANPAA
BV898_16261	49	4.19e-20	KGKTIRSEYE VQGDQLIKTYKTGDIVAKKWF KKVANPTEAP
BV898_16262	140	4.19e-20	KGKTIRSEYE VQGDQLIKTYKTGDIVAKKWF KKVANPTEAP
LOC129592137	140	2.07e-19	YNKTIVDIWH VDGDELVKTFKVAQVVAKRWY QRL
LOC129592129	139	2.62e-19	KDKVVHDTYH VEGEQLVKTYKVDNVEAKRWF AKATSKPSNT
BV898_16257	131	9.33e-19	RGKEVTEVYE VTGDELVKTYKIDGIVAKRYL KRQAV
BV898_16256	131	9.33e-19	RGKEVTEVYE VTGDELVKTYKIDGIVAKRYL KRQAV
H.Henanensis.Chr1.224	134	4.84e-18	KNKEIHDVYE VTGDELVKTCKVDGIVAKRSF KQESL
LOC129594944	143	1.70e-17	KGVTIHDVYH VEGEELVKSYTVGDVKAKKWF KKAVSKPANA
BV898_16255	145	3.82e-17	KTVSDSYHVF QNGTELEKTYKTGDTVAKRWY KKVISCH
BV898_00494	126	4.22e-17	KNLILHNVFA AQGDVLIKTYRVGNVVAKSWY RRLSSTADSN
LOC129595487	125	5.68e-17	PDRQVDQMFT FSGNEMVKTYKVGDVTAKVWF TKV
LOC129596311	144	4.26e-16	KNSKFHDKYH VVGDELVKTFTRGTWEAKRWF RRTSPSPVKS
RvY_02430	149	8.12e-16	LDKTINNEYK VEGNQLVKTSTLEGVTHKRYY NKRN
RvY_02424	134	2.00e-15	HNKVIHNTYK MDGQNLTKTSECEGVVHKRWY NKQQN
RvY_02431	150	4.05e-15	LDKTINNVFK LEGDHLVKTSTIEGVTMKRHY NKRQ
RvY_02618	85	7.43e-15	HNKVIHNTYE MDGQNLAKTSECEGVVHKRWY NKQQN
RvY_02437	99	8.81e-15	LDKTINNVFK LEGDHLVMTSTIDGVTMKRYY KTRT
H.Henanensis.Chr4.2190	117	1.24e-14	TGQHLHNIFD AQGDELIKILTVDGAVTRRWY RRCIASEIPD
RvY_03768	118	2.05e-14	LNQKFNEWCK VEDGELVKVFEGDCVTAKRWY TRTSGLPSS
BV898_01322	131	2.05e-14	IGKQYNENYE VVGQELIITYKMDGIVAKRFL KRDQSS
RvY_02365	146	2.23e-14	KNQETIVTYT ATGDDLEQTFTSNGVTGKRWY KKIHA
RvY_01561	121	4.31e-14	NNKHIKHSYK TEGDGYIKEYKTGNVMAKVWY KKSQQ
RvY_02438	147	4.68e-14	NNATYKNEFK VEGDFLVKTSDAHGIVHKRYY KRRN
LOC129584351	145	1.05e-13	KNKELKECYN VEGDKIVKNYESGNVKAKRIY KRVQN
LOC129590623	149	2.70e-13	KNIVLEEIYN VQGDKIMKEYVSGDIRAKRIY RRMNHL
RvY_02421	145	4.29e-13	KNTEMTHTYK VTGDELEHIFTSNGATGKKWY KKVNNAV
BV898_07130	129	1.79e-12	DGREVTHTYY PNADGFVKQFQLKDVIAKVWF KKDSA
BV898_19308	42	2.07e-12	DGREVTHTYY SNADGFVKQFQLKDVIAKVWF KKDSA
H.Henanensis.Chr5.2097	94	3.99e-12	TGKAWTETYE VKGKEMIKTFKIGSLVAKIFH QKKA
RvY_02420	139	6.14e-12	RNLTIFDTYR VNGTEMEVHSKVGDIESTRWY KKDIQP
LOC129602589	166	1.16e-11	NPLPIEHLYT SSSEGFILTYKIGDMIAKRSY KRV
H.Henanensis.Chr2.935	172	2.02e-11	NPMQIQHTYA ATDEGFTLIYKLGDVVAKSYY KRVSASNPVQ
H.Henanensis.Chr5.585	47	2.48e-11	INRMYEEVYE IKGDEMIKTITVAGVVGRTYL KKIPK
LOC129586548	111	4.89e-11	TNLETTAERE FTGDELITTLRAGDVTSVRKY KKVVP
H.Henanensis.Chr1.223	134	9.51e-11	KNKEIDYTYV VTGNELTKTYKDRDGVVAKRF YKQESL
RvY_02432	89	3.46e-10	LNITMHNVYK LEGDRLLKSSTIDGVTLNCHH KRRI
LOC129602685	111	3.69e-10	DGNFIEYVRE LKGDRLYLELKRGPILARRCF KKVPGL
H.Henanensis.Chr1.2239	129	1.44e-9	DGRDVTHTYT PENGGFVKQFQLKDVIAKLNF PESISNCTFP
LOC129582224	382	2.09e-8	YGKRIRQVTD EKFTEHQDRYKIGDIVVARWK GQENKPSVII

Motif Analysis_CAHS_Logo 1

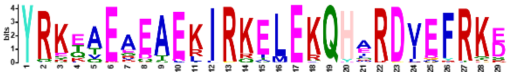

Name	Start	*p*-value	Sites		
LOC129588590	175	9.86e-34	EKELEKKTES	YRKQAEAEAEKIRKELEKQHARDVEFRKD	ILETTIDRQK
LOC129585805	121	9.86e-34	EKDLERKTEA	YRKTAEAEAEKIRKELEKQHARDVEFRKD	LVDTSIQRQK
H.Henanensis.Chr4.1171	126	9.86e-34	EKDLEKKTED	YRKTAEAEAEKIRKELEKQHARDVEFRKD	LVESAIDRQK
H.Henanensis.Chr1.1812	160	9.86e-34	EEEVARKTEA	YRKTAEAEAEKIRKELEKQHARDVEFRKD	LVESAIDRQK
H.Henanensis.Chr1.1811	129	9.86e-34	ERDLEKKTEA	YRKTAEAEAEKIRKELEKQHARDVEFRKD	LIESTIERQK
BV898_02951	124	9.86e-34	DKDLEKKTEE	YRKTAEAEAEKIRKELEKQHARDVEFRKD	LVESAIDRQK
BV898_04554	34	9.86e-34	EREMEKKTEA	YRKTAEAEAEKIRKELEKQHARDVEFRKD	LIESTN
BV898_04552	119	9.86e-34	EREMEKKTEA	YRKTAEAEAEKIRKELEKQHARDVEFRKD	LIESTIDRQK
BV898_10309	126	9.86e-34	EREMEKKTEA	YRKTAEAEAEKIRKELEKQHARDVEFRKD	LIESTIDRQK
H.Henanensis.Chr1.1813	129	3.24e-32	LSETERKTEA	YRKTAEAEAERIRKELEKQHARDIEFRKD	LVEGTIQTQK
BV898_04447	40	8.32e-32	EEEVAKKTEA	YRKTAEAEAEKIRRELEKQHARDVEFRKD	LVESAIDRQK
BV898_04448	109	8.32e-32	EEEVAKKTEA	YRKTAEAEAEKIRRELEKQHARDVEFRKD	LVESAIDRQK
BV898_04449	126	1.42e-31	QSETERKTEA	YRKTAEAEAERIRKELEKQHERDIEFRKD	LVQGTIDSQK
LOC129582959	178	2.14e-31	EKELEKKTDS	YRKEAEEEAEKIRKELEKQHARDVEFRKE	MVDTTIERQK
BV898_05814	188	6.13e-31	DKDLARKTEK	YQKEAEAEAEKIRKELEKQHARDVEFRKD	LVETAIDRQK
LOC129598089	127	1.42e-30	ERELEKKTEQ	YRKEAEAEAEKIRKELEKQHERDIEFRKG	LIDSAIERQK
H.Henanensis.Chr3.1778	185	9.85e-30	EADLAKKTEK	YQKEAEAEAEKIRRELEKQHARDVEFRKD	VVESTIDRQK
RvY_12093	110	1.31e-29	EKELEKKTEK	YRKEAEEQAEKIRKELEKQHHRDIEFRKE	VVESAIDRQK
LOC129602620	142	1.31e-29	ERAVEKMNEE	YRKKTEQEAEKIRKEMEKQHERDIEFRKE	LMEKAIERQK
RvY_00868	125	1.73e-29	EKEIARRTEL	YRKEQEAESEKIRKELEKQHHRDVEFRKE	LVEAAIEQQK
RvY_13011	189	2.99e-29	DKDLEKKTEA	YRKEAEHEAEKIRKALEKQHERDIEFRKE	VVGSTIEKQK
H.Henanensis.Chr4.1943	16	5.10e-29	EKELERKTEA	YRKQQEVETEKIRKELEKQHLRDVEFRKE	LMEQAIENQK
BV898_07587	122	5.10e-29	EKELERKTEA	YRKQQEVETEKIRKELEKQHLRDVEFRKE	LMEQTIENQK
H.Henanensis.Chr1.1742	119	7.55e-29	EKELERRTEI	YRKQAEQESERIRKELEKQHLRDIEFRKE	LADVAIENQK
BV898_12322	120	1.11e-28	EKELERRTEI	YRKQAEKESELIRKELEKQHMRDVEFRKE	LADVAIENQK
LOC129593136	127	1.63e-28	EKEVERKTEA	YRKLQEAEADKIRKELEKQHLRDVEFRKD	MVEMAIDNQK
LOC129592981	123	2.09e-28	EKEVEKKTEA	YRKQQEIEAEIIRKELEKQHIRDVEFRKE	LVEHAIENQK
RvY_18645	125	2.68e-28	ERELERKTEL	YRKQQEVETERIRKELEKQHLRDVEFRKE	LVEQTIEQQK
RvY_14505-1	101	3.89e-28	EKDLEKKTEA	YRKEAEAEAEKIRKQLEAQHARDVEFRKE	IVETAIDRQK
RvY_00946	115	1.15e-27	QRDIEGKTEA	YRKQAEQEAERLRKELEKQHQRDIEFRKS	LVQGTIENQK
RvY_01096	128	1.29e-27	EKDLEKKTEH	YRKEAEAEAERIRKALEQQHARDVEFRKE	VVESAIDKQK
RvY_00944	132	1.63e-27	EATLQKKAEE	YRHQTEAEAEKIRRELEKQHERDIQFRKD	LIDQTIEKQK
RvY_03957	121	2.31e-27	QKDIEGKTEA	YRKQAEAEAERLRRELEKQHQRDVEFRKS	LVQGTIDNQK
RvY_03280	157	2.59e-27	EKKLEKLTDS	YRKSTEEEAERIRKELEKQHERDVAFRKD	VLNSTIDRQQ
H.Henanensis.Chr2.1609	164	3.65e-27	DKHLEKVTDE	YRKKTEAEAEKIRKELEKQYKRDIDYRKE	VVEESVKRQK
BV898_04607	170	3.65e-27	DKHLEKVTEE	YRKKTEAEAEKIRKELEKQYKRDIDYRKE	MVDESVKRQK
LOC129594970	125	1.25e-26	KHALEAKTEA	YRKEAEQQAEKIRKEMEKQHERDVGFRQE	LVSETIAAQK
LOC129602618	151	1.39e-26	EKEVEKLTEK	YRRKTEAEAEKIRKELEKQHARDVEFREK	LVQEAVARQK
RvY_00945	160	8.56e-26	DHKMEKKAEK	YRKEAEKEADKIRKLMEKQHEKDVEFRKE	LVETSIEKQK
LOC129587456	420	1.30e-25	EMDTAQKAAM	YRSQVEADAELIRRTLERQHIRDIEFRKD	MVESAVDRQQ
H.Henanensis.Chr3.1042	309	1.95e-25	EEETSRKAAA	YRSEVEADAELIRQTLERQHMRDIEFRKD	MVETAVDRQQ
RvY_16825	407	1.74e-24	ERETGLMAAE	YRNEVERDAELIRQILERQHIRDLEFRKE	MIEKQVNRQE
RvY_12773	284	2.11e-24	ERDAGKRAAM	YREEVERDAELIRQILERQHIRDLEFRKE	MIEHQVNRQE
RvY_16236	184	2.11e-24	EQDAGMRAAQ	YREEVERDAELIRQILERQHIRDLEFRKE	MVENQVNRQE
H.Henanensis.Chr4.1944	126	3.40e-24	ARETQRQTEK	YRREAEEKAEKIRKELEEQHAKDIEFRKS	LVQQTVDTQK
LOC129595177	250	5.46e-24	EQDLMKRSEK	YQSETEAEALKIRKEMEKQHERDIAFRKS	LIDSAVDRQI
RvY_07296	212	3.79e-23	EEHMAKVTGK	YLEKTEHEAERIRKELEKQYKRDIDYRKE	LVEDSVKRQK
LOC129595015	73	5.42e-23	KHALKAKTEM	YRTEAEQQAEKIPKEVEKQHGRDVGFRQD	LVSETIAAQK
LOC129600971	131	8.47e-23	EKEIAKLTED	YREKTEKETAKIRKEMEKQHERDVEFRSK	LVEDAIKRQK
RvY_16824	291	1.71e-22	EREAGLRAAE	YRTEVERDAETIRQILERQHIRDLEFRRE	MIEHQVDRQE
H.Henanensis.Chr3.2436	111	4.83e-22	EKDVEKKTDS	YRKEAEEQTEKIRKEMEKQHQRDIERTTS	GATTISQHTE
LOC129592102	245	2.07e-17	DMELEKKTDS	YRKDAKEEAELIRKELEKQDERPKKIFGK	AIGRHPLEQE

Motif Analysis_CAHS_Logo 2

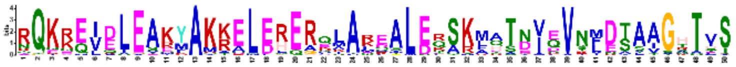

Name	Start	*p*-value	Sites		
BV898_02951	160	2.00e-50	RKDLVESAID	RQKREVDLEAKYAKKELEHERELAMNALEQSKMATNVQVQMDTAAGTTVS	GGTTVSEHTE
H.Henanensis.Chr4.1171	162	1.06e-48	RKDLVESAID	RQKREVELEAKYAKKELEHERELALNALEQSKMATNVQVSMDTAAGTTVS	GGTTVSQHTE
H.Henanensis.Chr1.1811	165	4.33e-48	RKDLIESTIE	RQKREVDLEAKMAKRELEREGQLAKEALERSRLATNVEVNFDSAAGHTVS	AGTTVSKSDK
LOC129588590	211	5.15e-48	RKDILETTID	RQKKEVELEAKMAKKELEHERQLALEALERSKMATNIEVNFESAAGRTVS	EGTVVSEHTD
BV898_10309	162	5.15e-48	RKDLIESTID	RQKREVDLEAKMAKRELDREGQLAKEALERSRLATNVEVNFDSAAGHTVS	GGTTVSTSDK
H.Henanensis.Chr1.1812	196	7.41e-47	RKDLVESAID	RQKREVDLEAKYAKTELEHERRLALEALERSKMQSNIEVNFDSAAGRTVT	ESHVVSEHSN
LOC129582959	214	1.65e-46	RKEMVDTTIE	RQKREVELEAKYAKKELEHERQLAQDALDKSRMATNIEVSMDTAAGRTVS	GGTTVSESVT
LOC129598089	163	5.76e-46	RKGLIDSAIE	RQKREVELEAKMAKRELEREAQLAKEALERSKLATNVEVNFDSAVGHTAS	VGTTVSESES
RvY_00944	168	1.44e-45	RKDLIDQTIE	KQKREVDLEAKMAKRELDREAQLAKEALERSRMATNVEVTLDTAAGHTVS	GGTTVSSVDK
H.Henanensis.Chr1.1813	165	2.26e-45	RKDLVEGTIQ	TQKKQVELEAMMAKRELDREAQIAKDALEQSKMATNVEVNFDSAAGHTVS	GGQTVSQSTK
RvY_01096	164	4.11e-45	RKEVVESAID	KQKREVDLEAKYAKKELEHERELAMNALEQSKLQTNVQVQMETAAGTTVT	GGTTVSQHTE
H.Henanensis.Chr2.1609	200	6.39e-45	RKEVVEESVK	RQKKELELEVKYAKKELDHERHLAKEALDQSKMRTDVQVNLDTSAGHTVS	GGSHVTEEEH
BV898_04448	145	6.39e-45	RKDLVESAID	RQKREVDLEAKYAKTELEHERKLALEALERSKLESNIEVNFDSAAGRTVT	ESHVVSQHTD
BV898_04607	206	2.35e-44	RKEMVDESVK	RQKKELELEVKYAKKELDHERLLAKEALEQSKMHTDVLVNLDTSAGHTVS	GGSHVTEEEY
RvY_14505-1	137	4.77e-44	RKEIVETAID	RQKREIELEAKYAKKELEHERQLALKALEQSKLATNVRVQMDTSAGSTFS	SGVSTHVVTN
RvY_07296	248	1.26e-43	RKELVEDSVK	RQKQELELEAKFAKKELERERELAKAALELSKMHTDVQVNMETAAGHTHS	NSHTSVQRTE
LOC129600971	167	4.64e-42	RSKLVEDAIK	RQKEELELEAKFAKKELERQRELALDALENSRMHTDISVNMDTTAGHTVS	SGHVDK
BV898_04447	76	8.81e-42	RKDLVESAID	RQKREVDLEAKYAKTELEHERKLALEPWNRSKLESNIEVNFDSAAGRTVT	ESHVVSQHTD
BV898_04449	162	8.81e-42	RKDLVQGTID	SQKKQVELGAIMAKRELDREAKLARDALEQSKMATNVEVNFDSAAGHTVS	GGQTVSQSTK
RvY_00946	151	1.00e-41	RKSLVQGTIE	NQKRQVELEAQLAKRELDREARLATQALDQSKMATDVQVNFDSAVGHTVS	GATTVSQSEK
BV898_05814	224	1.00e-41	RKDLVETAID	RQKQEIDLEAKKAKADLERERQMAKEALDNSKMQTNIEVQMNSAAGMTTS	GGTSVSESHV
LOC129585805	157	1.88e-41	RKDLVDTSIQ	RQKQEVELEARLAKKELEREAELAKEALEKSKLATNIEVKFDSAIGHTES	AGTTVSQSES
RvY_13011	225	2.14e-41	RKEVVGSTIE	KQKAEIELEAKRAKAALEHERQLANDALERSKMHTDVQVTMDTAAGHTVS	GGTTMSSSEQ
RvY_00945	196	9.45e-41	RKELVETSIE	KQKKEIEIEAKHAKAELEHERQLALEALERSKMTSNVEVNFDTSVGQTVS	ESHVGTHRTD
BV898_07587	158	1.21e-40	RKELMEQTIE	NQKRQIDLEARYAKKELERERNKVKRVLERSKFHTDIQVNMEAAAGSTHS	GSSSVAVSES
H.Henanensis.Chr1.1742	155	1.74e-40	RKELADVAIE	NQKKQIDIESRYAKRELDRQRQMARESLERSKFQSEIQVNLDTAAGATQS	GSSTIAQSES
RvY_18645	161	2.81e-40	RKELVEQTIE	QQKKMIDLEARYAKKELERERQKARQTLERAKFHTDIQVNMDASAGNTQS	SSSTVSQSEK
LOC129594970	161	4.03e-40	RQELVSETIA	AQKKQVDLEAQMAKKQLEREGQAAREALEKSKMTSNVEVNFDTAIGHAVS	GSTTVESEVN
RvY_03957	157	1.31e-39	RKSLVQGTID	NQKKQVELEAMMAKRELDREAKLAQDALEKSKMATDAQVTFESAVGHTIS	GSTTVSQSEK
H.Henanensis.Chr3.1778	221	2.09e-39	RKDVVESTID	RQKQEIDLEAKKAKADLERERQMAREALDSSKMHTDIRVQMNSAAGMTES	GGTTVSQSES
LOC129602618	187	3.32e-39	REKLVQEAVA	RQKEEILLEAKYAAKELERQRTLAMEALERSRHHANIQVNLDTVAGHTVS	ETQNIVTHYQ
BV898_12322	156	1.16e-38	RKELADVAIE	NQKKQIDIESRYAKRELDRQRQMARESLERSKFQSEIQVNLETAVGATQS	GSSTVAESEA
LOC129602620	178	1.65e-37	RKELMEKAIE	RQKEEIALEAKYARKELERQREMAMEALDKTKKQADVQVNLDTMAGHTVS	ESQSQSQQME
RvY_00868	161	1.90e-36	RKELVEAAIE	QQKRQLDLESRYAKKELDRQRQMARQALEHSKFDQKIEVTLDSAAGQTHS	HGETHSESEK
LOC129587456	456	2.88e-36	RKDMVESAVD	RQQHEIQLEAEYAMRALEKEREAARQALEQAKMETHIDVKVDSAIGTTIS	KGEVQTAAGR
RvY_16236	220	6.55e-36	RKEMVENQVN	RQEREIQLEAEYAMRALELERNAAKEALESAKAQTNVNVKVESAIGTTVS	KGAIQTSADK
RvY_16824	327	8.04e-36	RREMIEHQVD	RQEREIQLEAEYAMRALEMERQAARKALETAKAQTHVDVRVDTAIGTTIS	KGAISTSAEK
RvY_12773	320	2.00e-35	RKEMIEHQVN	RQEREIQLEAEYAMRALELERKAAREALEEAKAQTAVDVKFDTAIGVTIS	KGSISTSAEK
BV898_09037	17	2.70e-35	RKDLVESSVD	RQQQEIRLEAEYAMRALEQERVNARAALDQAMASTNIDVNIDSAIGTTHS	QGRVTTTSES
H.Henanensis.Chr3.1042	345	3.30e-35	RKDMVETAVD	RQQQEIRLEAEYAMRALEQERANARAALDRAQASTNIDVNIDSAIGTTHS	QGRVTTTSET
RvY_12093	146	4.91e-35	RKEVVESAID	RQKREVELAADYAKKRLEHEKALAMDALEQSKLRTDIQVNMNTATGSTST	GATVRSEHTE
RvY_03280	193	1.60e-33	RKDVLNSTID	RQQKEVDLQVKYAKKQLEHEREMAIHALEEAKAQAEVHVQMDTAAGTTFS	DVNSSASETP
RvY_16825	443	5.39e-33	RKEMIEKQVN	RQEEEIQLETEYALRALELERQAAREALETAKAQTHVDVRVDTAVGTTIS	RGDIDTHAER
LOC129592981	159	9.38e-33	RKELVEHAIE	NQKRQIDIESRYAKKELERERAKARMMLEKQKFHSDISVNLESHAANTQA	GEQLQVVSES
LOC129595177	286	1.95e-32	RKSLIDSAVD	RQIREVDLEARMAKRELLREAQIAKEQLDRASAATTVEVDLKTAVGHTHS	AGVTASETVM
LOC129593136	163	2.14e-32	RKDMVEMAID	NQKRQIDLESRYAKKEMDRERTRCRLMLEQQKFHSDISVNLEASAAHTET	SGQCVSESQK
H.Henanensis.Chr4.1944	162	3.36e-32	RKSLVQQTVD	TQKKQIDVEEKFAKKELERERLIAESALDQSRLQTVAQVSLDTAAGRATS	SGTVVSQSEK
H.Henanensis.Chr4.1943	52	1.29e-31	RKELMEQAIE	NQKRQIDLEARYAKKELERERNKARRVLENSKFHTDIQVIIVCLTSAQTQ	TQTQTYTIAA
BV898_07586	74	2.45e-24	QTDQYRKEMK	ETRKQIDVEAKFAQKELDREGRIAESALDQSRLQTIAEVSLERVTTKAIS	SGTIVSEKVT
LOC129592102	275	5.44e-21	ERPKKIFGKA	IGRHPLEQETKYAKKEQQREHHLTEESLDKSRVATNIDASMDAFASGSAR	TVSKPVNTSE

Motif Analysis_MAHS_Logo_1-3

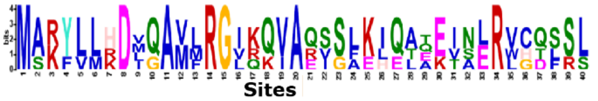

Name	Start	*p*-value	Sites		
BV898_03788	1	2.19e-47		MAKYLLHDMQAMLRGIKQVAQISLKIQAAEINERVCQSSL	RPRWSNLASS
H.Henanensis.Chr6.1494	1	2.71e-43		MAKYLLHDIGAMMRGIKQIAQVGFKIQAQEINERVCQSSL	LRSLTQQGNS
LOC129591308	1	2.23e-30		MARFVMKDLQAVFRGLQQVARSSAEHQLTETALRWHTLSL	RPLLQECVNK
RvY_05157	1	2.43e-28		MSRYLLRDVQAVLRGVRKVAESSLKLETEKVSLRLGDFRS	QPSLRSVPAS

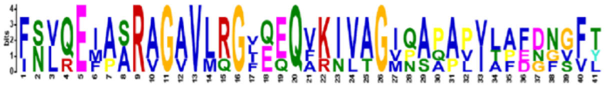

Name	Start	*p*-value	Sites		
BV898_03788	65	1.52e-47	PASSSSPRSS	FNVQEIASRAGAVLRGLEEQVKIVAGIQAPAPILAFDNGFT	LYSDKIGSAQ
H.Henanensis.Chr6.1494	65	9.34e-45	SSTKPSTFQG	FNVQEMASRAGAVLRGFEEQAKIVAGIQAPAPILAFDNGFT	LYSDKIGGAH
LOC129591308	59	3.44e-29	NKAQRSRTSG	ISLREFPARVGAVMQGIQEQVKILAGMPSQALVTPEGFVFY	TDKLDKDTHK
RvY_05157	58	2.34e-28	PASLTSRSQA	FSLQEIAARAGVVLRGVQQQFRNVTGVNAAPVVAFDNGSVL	YSERIHSQSS

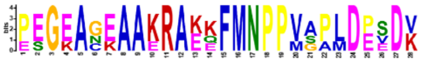

Name	Start	*p*-value	Sites		
BV898_03788	137	2.99e-31	DIDDENGHGK	PEGEAGKAAKRAEKFMNPPVAPLDESDV	SVLANNSLEG
H.Henanensis.Chr6.1494	137	3.51e-30	EASDENGNSL	PEGEAGEAAKRAEKFMNPPVGALDESDV	AHGEENAASL
RvY_05157	127	7.10e-28	PTGSVSNSPQ	PEGKANEAAERAKQFMNPPVAPMDPVDK	NEFVAMPEMG
LOC129591308	129	3.01e-27	HSAKVHGLKP	ESGEACEAAKRAKEFMNPPMSPLDPEDK	NEVVRTPEMS
